# Minimum data requirements and automated preprocessing for reliable EEG biomarkers in Rett syndrome

**DOI:** 10.3389/fneur.2026.1791834

**Published:** 2026-06-16

**Authors:** Yongtaek Oh, Kathleen Campbell, Justine Shults, Joni Saby, Eric D. Marsh

**Affiliations:** 1 Division of Neurology, Children's Hospital of Philadelphia, Philadelphia, PA, United States; 2 Division of Developmental and Behavioral Pediatrics, Children's Hospital of Philadelphia, Philadelphia, PA, United States; 3 Department of Neurology, University of Pennsylvania Perelman School of Medicine, Philadelphia, PA, United States; 4 Department of Biostatistics, Epidemiology and Informatics, University of Pennsylvania Perelman School of Medicine, Philadelphia, PA, United States; 5 Department of Pediatrics, University of Pennsylvania Perelman School of Medicine, Philadelphia, PA, United States

**Keywords:** automated preprocessing, biomarkers, data sufficiency, EEG, Rett syndrome

## Abstract

**Background:**

Electroencephalography (EEG) is a promising biomarker for Rett syndrome (RTT), but excessive artifact and variable tolerance for longer recording sessions pose challenges for reliable biomarker development. Establishing an automated preprocessing pipeline that matches human review and defining the minimum data needed for stable quantitative EEG (qEEG) features can support more patient-friendly protocols and provide consistent multisite analysis results.

**Methods:**

A mean of 10 min of resting-state EEG from 117 participants (1–18 year old; 236 sessions) in the multisite R61 RTT study was processed using a fully automated, correction-based preprocessing pipeline incorporating artifact handling, adaptive channel rejection, ASR, and ICA-based cleaning. Spectral power was extracted from artifact-free 4-s epochs. The proposed pipeline is validated using an established rejection-based pipeline. Feature stability as a function of cumulative data length was then assessed using two complementary frameworks: a Statistical Convergence approach and a Model-Based Inflection approach, and potential systemic dependencies were evaluated using permutation analyses. The relationship between clinical measures was also assessed.

**Results:**

The correction-based pipeline retained substantially more data than the rejection-based workflow (mean retention = 95.0% vs. 28.4%; *p* < 0.001) while preserving strong feature correspondence across frequency bands. Stable power estimates were achieved after 19–34 epochs (= 76–136 s). Based on permutation analysis, there was no statistically significant difference in minimum stabilization threshold between RTT and TD. However, the RTT group exhibited higher rates of intrinsic signal instability than typically developing (TD) controls. Age-stratified analysis revealed that the minimum epochs did not significantly differ between age groups. Spectral associations with clinical severity were preserved when using only the minimum data required for stability, as well as in an ecologically valid scenario of truncating the raw EEG up to minimum epoch recommendation and reprocessing it.

**Conclusions:**

With the proposed correction-based pipeline, approximately 3 min of raw resting-state EEG are sufficient to obtain stable and clinically meaningful spectral features in children with Rett syndrome. These findings support shorter, more feasible EEG acquisitions and provide a reproducible framework for data sufficiency in multisite neurodevelopmental studies.

## 1 Introduction

Rett syndrome (RTT) is a severe neurodevelopmental disorder primarily caused by loss-of-function variants in the MECP2 gene. Affected individuals experience an early developmental regression characterized by loss of purposeful hand use and speech, emergence of hand stereotypies, motor dysfunction, autonomic irregularities, and cognitive impairment (1). Despite well-defined genetic and clinical features, objective biomarkers that reliably capture underlying neural dysfunction and treatment effects remain limited.

Electroencephalography (EEG) has emerged as a promising biomarker for RTT, offering a direct, non-invasive measure of brain function that can reflect excitatory–inhibitory balance, connectivity, and neural maturation (2–4). Quantitative EEG (qEEG) features have been increasingly explored as outcome measures in RTT natural history studies and early-phase clinical trials, providing a scalable tool for evaluating treatment response and disease progression (5, 6). Across these studies, EEG features such as spectral powers, aperiodic activity, and power ratios have been shown to correlate with severity of RTT-related symptoms and/or disease progression.

Unfortunately, recording high-quality EEG in participants with RTT can be challenging. Participants with RTT often show variable tolerance for EEG procedures, including difficulty remaining still, agitation during net/cap placement, and shortened recording durations due to behavioral challenges (7). These limitations result in challenges for downstream analyses, including the potential for substantial data loss. Without data-driven standards for the minimum recording duration required for stable feature estimation, studies risk excluding valuable data or overinterpreting unstable measures. Addressing this gap is critical to advancing EEG as a reliable biomarker in RTT, ensuring both methodological rigor and inclusivity of participants for long recordings.

In this study, we define feature stability as the point during an EEG recording at which additional artifact-free data no longer meaningfully alters the extracted features or reduces their variability. Once this point is reached, adding more data provides little additional information about the underlying neural signal. Identifying this stability point provides an evidence-based threshold for how much data is “enough” for valid spectral analysis—a question central to improving the reliability, efficiency, and feasibility of EEG biomarkers in RTT and related neurodevelopmental disorders. This consideration is especially critical for children with RTT and other neurodevelopmental conditions who may not tolerate long recording sessions, making it essential to determine the minimum recording length that still yields reliable features.

Previous work has examined data sufficiency in EEG, but primarily outside the context of rare neurodevelopmental disorders in children. Early adult studies reported that 40 to 60 s of artifact-free data is required to minimize variability in spectral estimates, often necessitating 6 to 8 min of raw recording to accommodate data loss from strict artifact rejection (8–10). More recent large-scale analyses have refined these estimates. For instance, Wiesman et al. (11) used MEG to analyze recordings from healthy adults and suggested that 30 to 120 s of artifact-free data is required to capture stable estimates across all frequencies, recommending up to 10 min of raw data recording to capture stable high-frequency characteristics. Similarly, Jin et al. (12) studied patients aged 50 to 90 with dementia or Alzheimer's disease and reported that 1 min of clean EEG data is sufficient for stable spectral representation, although 30 min of raw EEG was recorded to ensure sufficient yield of clean EEG data. Pediatric neurophysiology research has also utilized thresholds comparable to those used in adult EEG studies. A survey of pediatric resting-state EEG methods from 66 self-identified experts revealed a lack of field-wide consensus regarding the minimum threshold for artifact-free data (13). While the reported mean cutoff for a stable, clean data was 52 s (SD = 49 s), the responses varied drastically, ranging from no minimum (1 s) to 210 s. This survey highlights significant heterogeneity in inclusion criteria and underscores the lack of dedicated studies examining the stability of pediatric EEG. Consequently, no study has evaluated the stability of power features in children with NDDs or specifically in Rett syndrome, where characteristic neurophysiological abnormalities and pronounced movement-related artifacts may substantially alter stability requirements. Moreover, most prior work relied on manual or heterogeneous preprocessing pipelines, limiting reproducibility and complicating translation to large-scale clinical datasets. These gaps highlight the need for a disorder-specific, fully automated, and statistically rigorous framework for defining minimum data requirements in RTT.

Accurate determination of feature stability requires standardized preprocessing to minimize variability introduced by subjective artifact rejection. Data cleaning can involve two dimensions: rejection vs. correction, manual vs. automatic. Past studies of EEG biomarkers in Rett syndrome have mostly adapted “rejection-based” approaches involving manual judgment including visual inspection of bad channels and/or segments followed by amplitude-based rejection techniques that relied on set-thresholds (5, 6, 14). This semi-automatic approach, though common, is time-intensive, susceptible to operator bias, and may lead to substantial data loss if the parameter is not optimized. To overcome these constraints, we implemented a fully automated preprocessing pipeline with a “correction-based” approach optimized for EEG recorded from participants with RTT, incorporating robust artifact handling methods such as bad-channel detection via Local Outlier Factor (LOF) with adaptive thresholding and Artifact Subspace Reconstruction (ASR). Although other automated EEG preprocessing pipelines have been developed for pediatric EEG (15, 16), these pipelines were not developed or tested specifically for children with severe genetic neurodevelopmental disorders (NDD) such as RTT. Given the prevalence of background EEG abnormalities common in these disorders, other pediatric EEG pipelines may perform less optimally with these groups, risking over-rejection of data or signal distortion (17).

To address these challenges, the primary objective of the present study is to establish quantitative recommendations for number of artifact-free epochs required for stable EEG spectral feature estimation in the context of EEG studies involving RTT participants. Because RTT EEG recordings are inherently artifact-heavy, calculating these minimum thresholds requires a pipeline that can maximize the amount of usable data. We therefore utilized the correction-based preprocessing pipeline. As a prerequisite, we first validated this correction-based workflow against the established “rejection-based” pipeline used in prior studies to verify improved data retention and spectral feature correspondence. Using the robust data yield from the correction-based pipeline, we then evaluated feature stability as a function of cumulative recording length via two complementary analytical approaches: (1) a non-parametric convergence method, which identifies the point at which feature variability becomes statistically indistinguishable from long-duration reference estimates, and (2) a model-based inflection method, which detects when the decline in variability reaches a stable plateau. Finally, we examined whether using only the minimum stable data preserved known associations between spectral power and RTT clinical severity. By establishing data-driven thresholds for minimum recording duration alongside a validated preprocessing workflow that maximizes data retention, this work aims to advance the reliability, feasibility, and translational utility of EEG biomarkers in RTT for future multisite research and clinical trials.

## 2 Materials and methods

### 2.1 Participants

EEG data were analyzed from participants enrolled in the R61 Rett Syndrome Study (NCT05932589) between 2023 and 2025. The dataset represents a multisite collection of EEG recordings obtained at Boston Children's Hospital, Children's Hospital Colorado, Children's Hospital Los Angeles, Children's Hospital of Philadelphia, Texas Children's Hospital, and Vanderbilt University Medical Center. All procedures were approved by the Institutional Review Boards of participating institutions, and written informed consent was obtained from all participants or their legal guardians in accordance with the Declaration of Helsinki.

**Figure 1:**
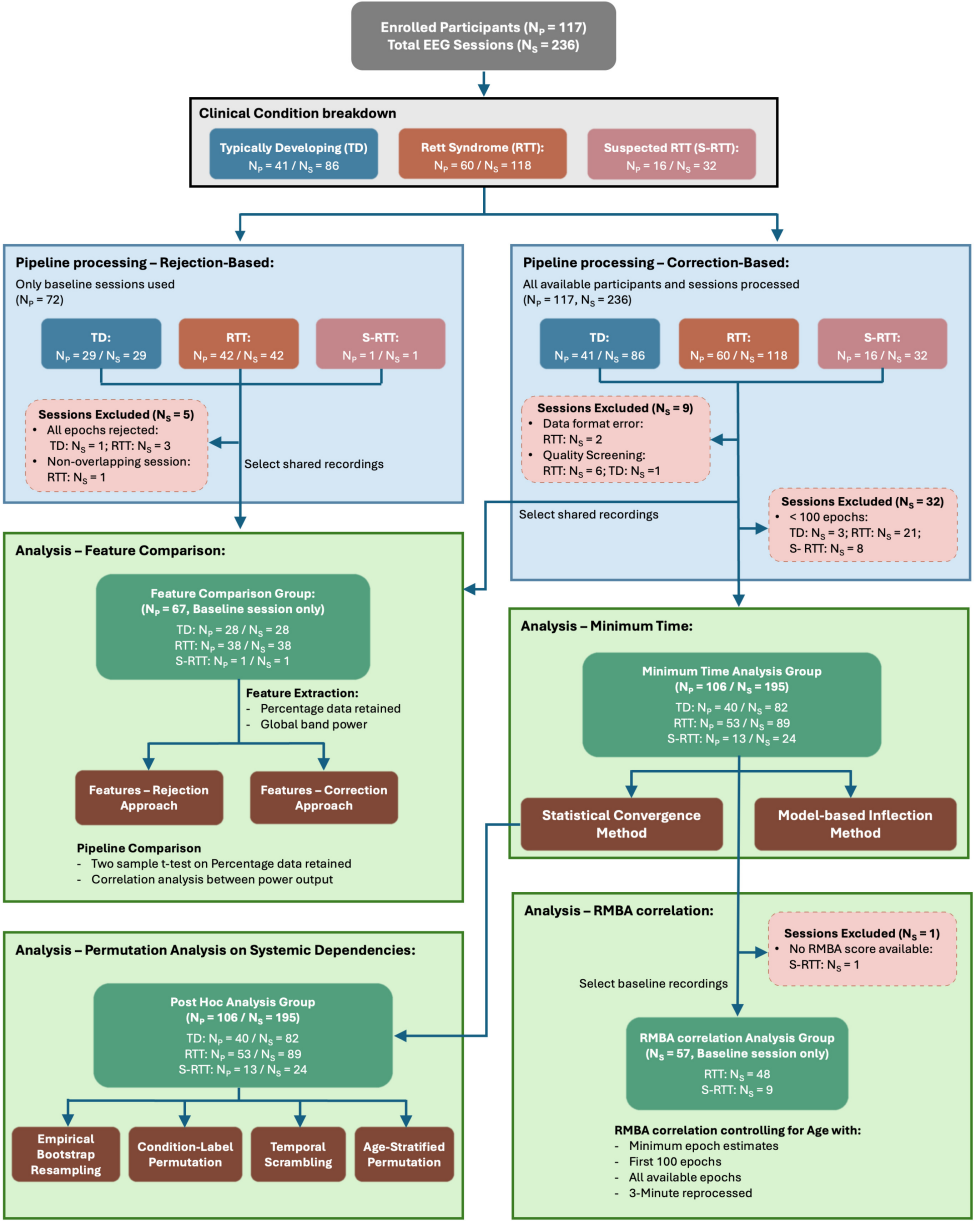
Flow of participants and recording sessions through processing pipelines, inclusion criteria, and analysis steps. The flowchart details the progression from the initial pool of EEG sessions to the final analytical samples. The top level (gray box) indicates the clinical condition breakdown of the available data, including the Typically Developing (TD), Rett syndrome (RTT), and Suspected RTT (S-RTT) cohorts. The subsequent level (blue boxes) outlines the two processing tracks utilizing different artifact handling approaches: the rejection-based pipeline and the correction-based pipeline. The inclusion criteria and the specific reasons for data exclusion are also detailed. Following the preprocessing pipelines, the processed EEG data is then allocated to four distinct analyses (green boxes). These tracks encompass the validation of the correction-based pipeline via feature comparison, the minimum time analyses (which incorporate the statistical convergence method and the model-based inflection method), permutation analyses of systemic dependencies that may influence feature stabilization, and the final RMBA clinical correlation evaluated using both the minimum number of epochs and the simulated truncated data. NP denotes the number of unique participants, and NS denotes the total number of recording sessions.

The participant flow, exclusion criteria, and overall analytical pipeline are summarized in Figure 1. A total of 117 female participants (mean age = 7.95 ± 4.60 years; range = 1–18) contributed 236 EEG sessions across four longitudinal time points: baseline, 2 weeks, 6 months, and 1 year. The cohort included 60 participants with a clinical diagnosis of Rett syndrome (RTT group; mean age = 9.40 ± 4.55; range = 3–18) contributing 118 EEG sessions, and 41 participants who were typically developing (TD group; mean age = 7.95 ± 3.92; range = 2–16) contributing 86 EEG sessions. An additional 16 participants were classified as “Suspected RTT” (S-RTT group; mean age = 2.50 ± 1.10; range = 1–5), and contributed 32 EEG sessions. Individuals in the S-RTT group exhibited Rett-like features but were below the diagnostic age threshold or had a pathogenic variant in MECP2 but lacked distinct RTT features. These individuals were included in the pipeline comparison but were excluded from RTT vs. TD group comparisons. For the RTT and S-RTT groups, clinical severity was evaluated using the Revised Motor Behavioral Assessment (RMBA) (18). When RMBA scoring and EEG acquisition did not occur on the same day, the closest available assessment was used.

### 2.2 EEG acquisition

EEG acquisition procedures and equipment were standardized across all participating sites following recommended multisite practices for genetic neurodevelopmental disorder studies (7). Recordings were obtained using 128-channel Geodesic Sensor Nets and Net Amps amplifiers (Magstim EGI). EEG was recorded with a sampling rate of 1,000 Hz and referenced online to Cz (corresponding to electrode E129 in the EGI system). This harmonized acquisition protocol ensured consistency in data collection across recording sites.

Each EEG session included three paradigms: Resting-State EEG (Rest), Visually Evoked Potentials (VEP), and Auditory Evoked Potentials (AEP), following procedures described by Saby et al. (19). The present study analyzed only the resting-state portion of each session. Rest recordings had a mean duration of 10.07 min (range = 2.45–17.01 min). During Rest and AEP paradigms, participants were permitted to view a silent movie to facilitate comfort and minimize movement. The order of VEP, AEP, and Rest tasks was randomized across sessions. Throughout each recording, trained research staff monitored participants to ensure wakefulness and sustained engagement with the task when applicable.

### 2.3 EEG processing

EEG data were preprocessed using two pipelines with differing approaches to handling artifacts: (1) a “Rejection-based” preprocessing workflow adapted from prior RTT and CDKL5 studies (5, 19), and (2) a “Correction-based” fully automated pipeline optimized for high-artifact pediatric EEG. The primary distinction in correction-based pipeline was the use of automated bad-channel detection, Artifact Subspace Reconstruction (ASR), and Independent Component Analysis (ICA) in the automated workflow. The purpose of the initial comparison was to validate the correction-based pipeline in terms of the replication of features compared to the rejection-based pipeline, which were used in the past published studies.

#### 2.3.1 Rejection-based preprocessing pipeline

**Figure 2:**
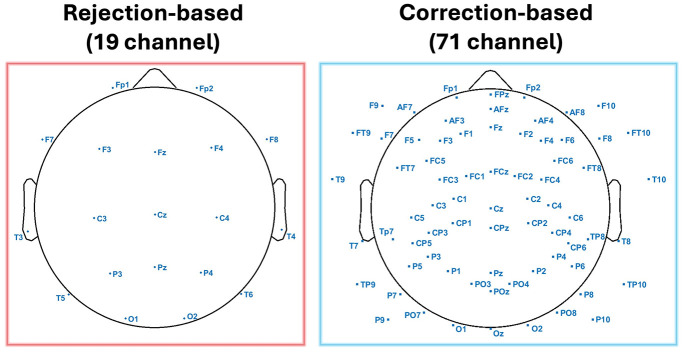
Channel montages utilized in the rejection-based and correction-based preprocessing pipelines. The red panel illustrates the channel configuration for the rejection-based pipeline, which employed a standard 19-channel 10–20 layout. These virtual channel locations were interpolated via spherical splines following manual bad channel rejection in BESA. The blue panel details the montage for the correction-based pipeline, which preserved the original recorded sensor locations. A subset of 71 channels was selected from the original 128-channel array based on technical guidelines identifying the closest structural analog to the standard 10–10 system (20). The topographical map on the right visualizes this specific 71-channel configuration relative to the full 128-channel layout, adapted from Luu and Ferree (20).

The rejection-based pipeline followed procedures described previously (5, 19). Because this pipeline served exclusively as a validation benchmark for the correction-based pipeline proposed in this work, a representative subset comprising the baseline sessions from 42 RTT, 1 S-RTT, and 29 TD participants was utilized. Notably, this participant pool was analyzed in a prior study that successfully identified qEEG features correlated with clinical severity scores (5), making it an ideal benchmark for validation. For these sessions, the sampling rate was maintained at 1,000 Hz. Initial visual inspection was conducted in BESA 7.0 (BESA GmbH, Gräfelfing, Germany) to identify and remove channels with excessive noise, high impedance, or flatlining. The number of rejected channels did not exceed 20% of the total channel count for any given session. To standardize the spatial layout across participants after channel rejection, the EEG data were spatially interpolated to obtain a 19-channel virtual montage based on standard 10–10 locations using BESA's spherical spline interpolation (Figure 2). The resulting 19-channel data were average-referenced and exported in EDF format for subsequent automated processing in MATLAB 2023a.

In MATLAB, data were spatially filtered using the Hjorth nearest-neighbor Laplacian (21) to improve spatial specificity, then processed with a 2–40 Hz band-pass filter and 60 Hz notch-filter, both using 4th order Butterworth filters. Artifact rejection was performed using a two-stage non-overlapping sliding window procedure: (1) windows with absolute amplitude exceeding 150 μV in any channel were removed, and (2) remaining windows were screened using RMS amplitude and line-length thresholds based on the median absolute deviation. Clean, contiguous windows spanning 4 s were retained as artifact-free segments for spectral analysis. The resulting data served as the reference for validating the correction-based pipeline.

Three RTT and one TD participant were excluded from the final group for the rejection-based pipeline because no usable data remained after artifact rejection. One additional RTT participant was excluded because the session from this participant did not overlap with the sessions remaining from correction-based pipeline output, resulting in a final rejection-based processed validation sample of 38 RTT, 1 S-RTT, and 28 TD participants (Figure 1).

#### 2.3.2 Correction-based preprocessing pipeline

**Figure 3:**
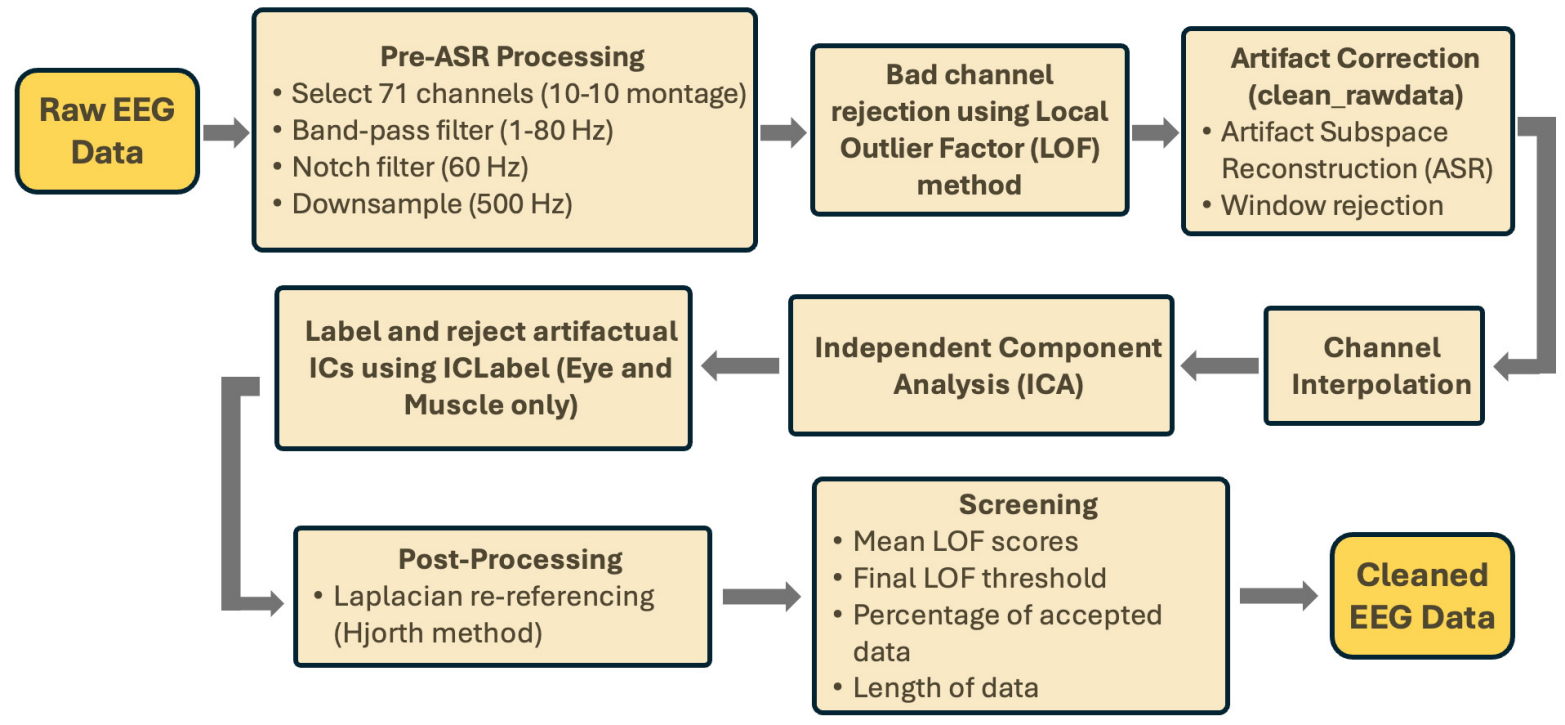
Workflow diagram of the correction-based EEG processing pipeline. The workflow diagram illustrates the sequential steps from raw EEG input through preprocessing, artifact rejection, ICA-based cleaning, post-processing, and final screening to generate cleaned EEG data.

The correction-based preprocessing pipeline was performed in MATLAB 2023a using EEGLAB (22) and associated plugins including clean_rawdata, NEAR_ChannelReject, ICLabel, and scd (pipeline workflow summarized in Figure 3). All 236 available sessions were processed using the correction-based pipeline. The code for the pipeline is available in GitHub repository (https://github.com/ytoh2000/Rett_MinimumTime).

The EEG was first reduced from 128 channels to 71 channels corresponding to the international 10–10 montage to standardize spatial coverage across recording systems based on the technical note provided by the manufacturer (Figure 2) (20). The selection of 71 channels allows exclusion of outer ring channels of EGI Net, which are more prone to muscle artifacts and poor contact in the pediatric population (15). Data were band-pass filtered (1–80 Hz) and notch-filtered at 60 Hz to remove slow drifts and line noise, then down-sampled to 500 Hz to reduce computational load while maintaining good temporal resolution.

Bad-channel rejection was performed using the Local Outlier Factor (LOF) method in NEAR plugin (23), which identifies channels with abnormal spatial-temporal characteristics relative to their neighbors. Default parameters were used (flatline threshold: 5 s, LOF threshold: 2.5), with an adaptive rejection cap of 10%, which constrains the maximum proportion of channels that can be rejected and automatically increases the LOF threshold if this limit is exceeded. Setting this rejection cap is particularly important in RTT studies, as RTT EEG often shows higher amplitude fluctuations and elevated low-frequency power relative to TD EEG, which could otherwise lead to over-rejection of clinically meaningful but atypical channels (5). For comparison, RANSAC (random sample consensus) -based spatial correlation method in clean_rawdata plugin (24) was applied in parallel. A default channel correlation threshold of 0.85 was used to reject poorly correlated channels. Rejected channels from both methods were interpolated using spherical spline interpolation.

Following channel interpolation, EEGLAB's clean_rawdata plugin was used to perform Artifact Subspace Reconstruction (ASR) and window rejection. ASR was applied to suppress transient high amplitude artifacts (25–27). ASR identifies a clean portion of the data to be used as calibration dataset and compares the covariance structure of the data within short sliding window. Windows or components whose variance exceeds a specified multiple of the baseline variance are considered “bursts” and removed. Then the remaining components are reconstructed using subspace projection, thereby preserving the underlying neural signal while removing artifactual transients. Optimized parameters (burst criterion = 25; window length = 0.5 s) were selected and used based on maximizing the known delta-RMBA association (see Supplemental material, Supplementary Figure 2). Following ASR, a final window rejection step was applied to remove any data segments where more than 25% of the channels still exhibited excessive noise.

To further reject artifactual non-neural sources, Independent Component Analysis (ICA) was performed, followed by ICLabel classification (28) to automatically categorize individual independent components. Components labeled as “eye” or “muscle” with probability over 0.75 were rejected. The data were then spatially filtered using the Hjorth nearest-neighbor approximation (21), matching the method used in the rejection-based pipeline. The placement of Laplacian step toward the end of the pipeline diverged from the sequence used in the rejection-based benchmark. This particular order of processing steps was methodologically necessary to preserve performance of Independent Component Analysis (ICA) and the associated scalp topographies for individual components. Automated component classification algorithms, such as ICLabel, are trained on ICA components based on the common average reference (28). Computing the Laplacian prior to ICA alters the resulting component topographies, which may significantly compromise classification accuracy. Applying the Laplacian strictly after ICA ensured optimal automated artifact removal while successfully achieving final spatial parity with the rejection-based benchmark data. Lastly, a low-pass filter at 50 Hz was applied.

After all processing steps, data quality of individual EEG was screened based on four metrics: (1) the mean Local Outlier Factor (LOF) score of retained channels, (2) the final adaptive LOF threshold, (3) percentage of accepted data (< 50%), and (4) total accepted data length. Outliers were identified relative to the cohort distribution using a 3-SD cutoff: recordings were flagged if their mean LOF score for good channels or their final adaptive LOF threshold exceeded the group mean +3 SD, or if less than 50% of data points remained after preprocessing, or if their accepted data length fell below the group mean −3 SD. Among 236 sessions, 2 were excluded due to error in the data format and 7 were excluded during the data quality screening, resulting in 227 sessions (Figure 1).

#### 2.3.3 Feature extraction

Spectral power features were extracted from artifact-free 4-s epochs using identical procedures across pipelines. Power spectral density was estimated using the periodogram method (Hamming window, no overlap) and averaged over canonical frequency bands: delta (1–4 Hz), theta (4–8 Hz), alpha (8–13 Hz), beta (13–30 Hz), and gamma (30–50 Hz). The PSD values were log-transformed. Extracted spectral power were used in two ways: (1) validation of correction-based pipeline via feature comparison from rejection-based pipeline, and (2) stability analysis, or determination of the minimum number of EEG data epochs. For validation of correction-based pipeline, spectral power features were averaged across epochs to produce one spectral feature vector per session. For stability analysis, the time-series of 4-s features was retained to assess how feature variability evolved with cumulative recording length.

### 2.4 Evaluation of correction-based pipeline

The performance of the fully correction-based pipeline was evaluated by (1) comparing percentage of retained EEG data, and (2) evaluating correspondence of spectral features with rejection-based pipeline. First, matching sessions from rejection-based and correction-based pipelines were identified, which included baseline sessions from 67 participants (Figure 1). A boxplot was generated to visualize the trend and distribution of the percent accepted data for each pipeline. Scatterplots with Pearson correlation coefficient and r-squared (R^2^) values were generated to visualize and quantify the agreement; differences in retained data between the pipelines were colorized to characterize systematic deviations.

### 2.5 Determining the minimum stabilization threshold

To quantify feature stability as a function of recording duration, we computed a modified coefficient of variation (mCV) across cumulative 4-s epochs for each participant and frequency band. At each epoch count *n*, spectral power values *x*_*i*_were aggregated sequentially, and mCV was defined as:

$$\begin{array}{l} \text{m}CV=\;\frac{SD}{MAD},\text{where}\;MAD=\frac{1}{n}\sum_{i=1}^{n}|x_{i}-\bar{\bar{x}}| \end{array}$$

Unlike the conventional coefficient of variation ($CV=\;\frac{SD}{Mean}$), the mCV replaces the mean in the denominator with the mean absolute deviation (MAD) from the mean. This adjustment prevents instability when log-transformed band-power means approach zero and reduces sensitivity to outliers. Normalizing SD by MAD therefore yields a stable and scale-independent measure of relative variability, capturing the consistency of oscillatory power irrespective of its absolute magnitude.

**Figure 4:**
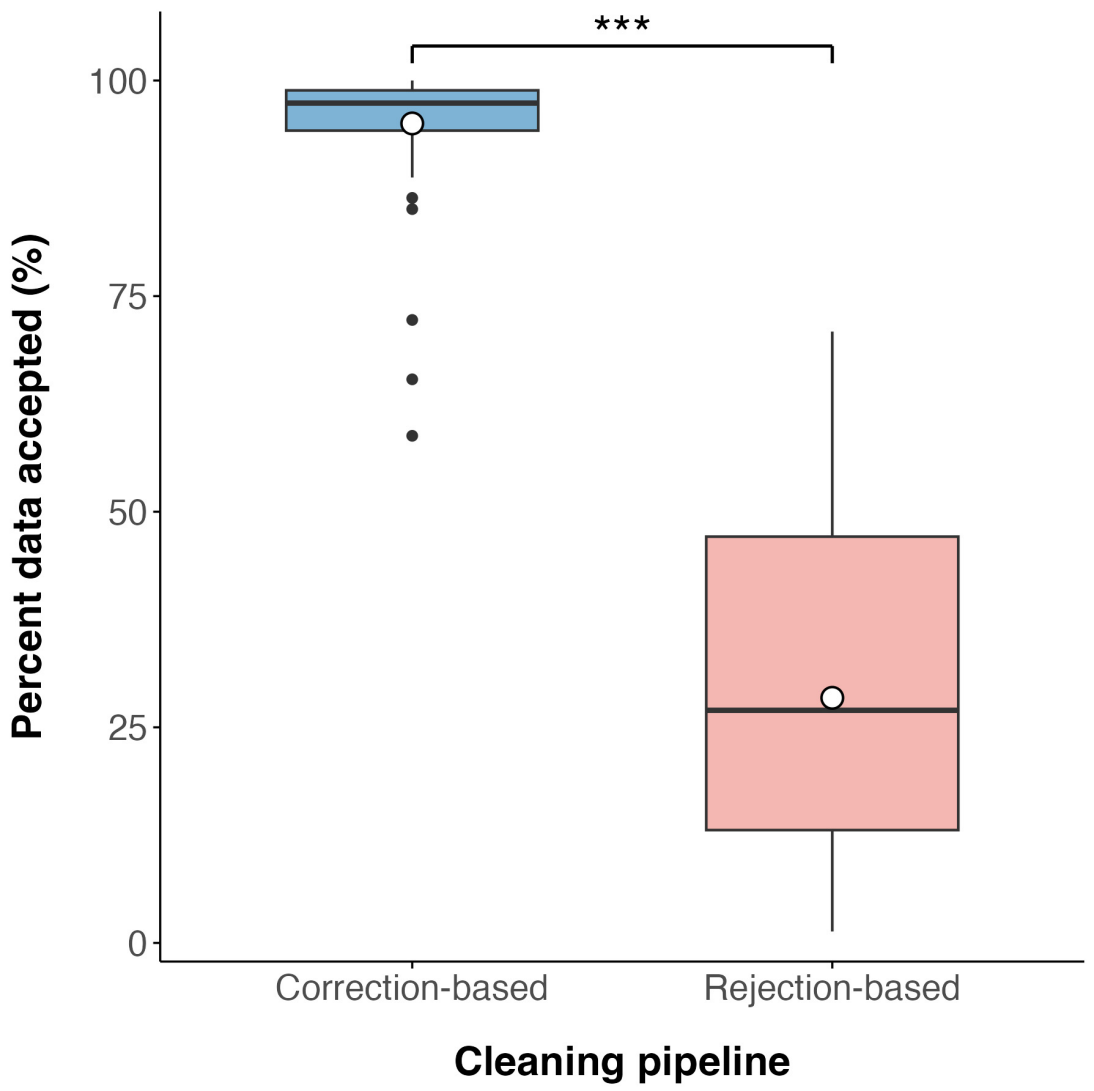
Comparison of data retention across cleaning pipelines. Boxplots illustrate the percentage of data accepted by the correction-based and rejection-based preprocessing pipelines. Within each box, the central horizontal line represents the median, and the box boundaries indicate the interquartile range. Whiskers extend to 1.5 times the interquartile range, and black dots denote outliers beyond this limit. The white dot marks the mean percentage of retained data for each method. The correction-based pipeline retained a substantially greater and more consistent proportion of data compared to the rejection-based pipeline. Statistical significance between the two pipelines was determined via a two-sample *t*-test and is denoted by asterisks (***p < 0.001). For the rejection-based pipeline, three Rett syndrome and one typically developing recording were excluded from this visualization because no usable data remained after preprocessing.

**Figure 5:**
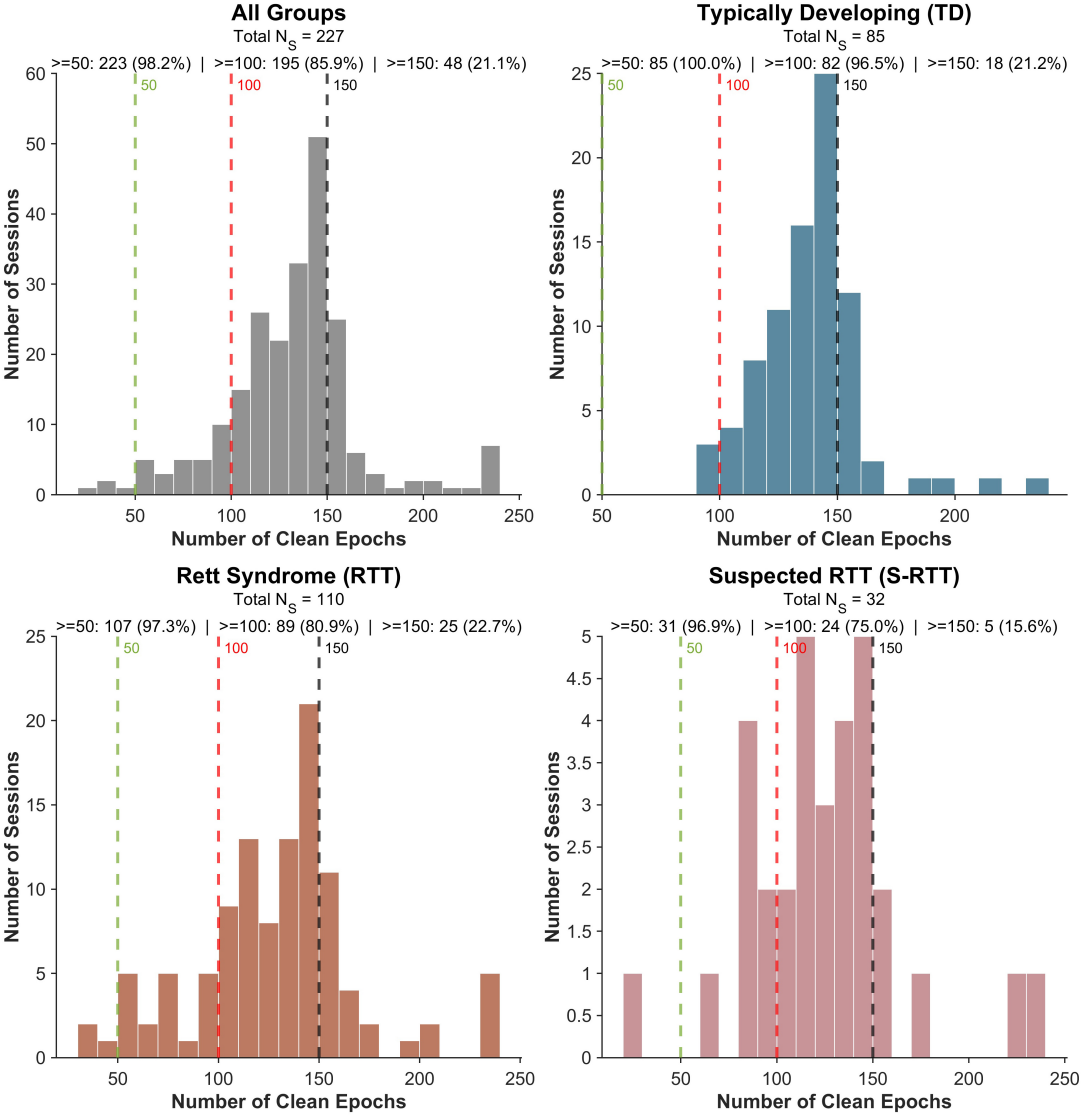
Distribution of retained data across all preprocessed EEG sessions using the correction-based pipeline. Histograms display the number of sessions based on the number of clean 4-s epochs retained after applying the correction-based pipeline. Distributions are shown for the entire combined cohort as well as individually for the Typically Developing (TD), Rett syndrome (RTT), and suspected Rett syndrome (S-RTT) clinical groups. Vertical dashed lines denote the epoch count cutoff at 50 (green), 100 (red), and 150 (black), with the corresponding session retention percentages detailed above each plot. A conservative threshold of 100 epochs (400 s), indicated by the vertical red line, was established to define the stable reference. This threshold functions as an empirical data-driven filter, effectively trimming the heavy left tail of the distribution to exclude low-quality outlier sessions while preserving the primary cluster of robust, high-yield recordings.

Only the data processed with the correction-based pipeline were utilized for the stability analysis. This was because the rejection-based workflow produced substantially lower and highly variable amounts of artifact-free data (Figure 4), making it unsuitable for an analytic approach involving set number of cumulative-epochs. To establish a reliable long-duration reference to assume stable feature estimate, a threshold of 100 clean epochs (400 s) was set as inclusion criteria. Consequently, an additional 32 sessions were excluded from the remaining 227 preprocessed recordings. The 100-epoch threshold was explicitly chosen as a conservative approach to double the maximum acceptable clean data cutoff (210 s) reported in the survey of pediatric EEG experts (13). Such conservative benchmark was necessary as RTT patients exhibit significantly higher variability in qEEG feature than the typically developing pediatric population (5). Furthermore, 100-epoch thresholding functioned as an empirical data-driven filter to remove the heavy left tail of the distribution, effectively identifying and excluding the bottom 15th percentile of low-quality outliers (Figure 5).

The resulting analysis cohort for the minimum analysis consisted of 195 sessions from 106 participants, comprising 53 RTT, 13 S-RTT, and 40 TD participants. To determine whether clinical diagnosis inherently affects the minimum data required for stability, the analysis was conducted on the combined cohort including all conditions, as well as separately for RTT and TD groups.

Two complementary methods were used to define the stability point: (1) a Statistical Convergence approach and (2) a Model-Based Inflection approach.

#### 2.5.1 Statistical convergence approach

For each interim epoch length, the modified coefficient of variation (mCV) values were compared with the reference mCV computed from the full 100 epochs using the Wilcoxon signed-rank test. This non-parametric test was selected for its robustness to the non-Gaussian distributions observed in the current mCV values. Stabilization threshold was defined as the first epoch at which the interim estimate was statistically indistinguishable from the reference (*p* > 0.1) and the corresponding Cohen's d was less than 0.1, indicating negligible effect size (29). To prevent spurious mathematical stabilization at extremely low volumes, the convergence analysis only evaluated cumulative length starting from 10 epochs (40 s). This absolute threshold served as a biological plausibility constraint supported by literature, aligning with minimum clean data thresholds in adult populations (9) and approximating the average acceptable clean data cutoff (52 s) reported in a recent consensus survey of pediatric EEG experts (13). To avoid spurious transient minima, a local-minimum detection algorithm with a 15-epoch lookahead window was applied, ensuring that no later segment produced lower variability. The analysis was performed using custom script in MATLAB 2023a.

#### 2.5.2 Model-based inflection approach

To characterize the overall trajectory of variability reduction, we modeled mCV as a function of epoch count using Generalized Estimating Equations (GEE) with an exchangeable working correlation structure and robust standard errors. Fourth-degree polynomial models were selected after sequential comparison of lower-order models using Wald tests and GEE goodness-of-fit criteria (30). The model fit is outlined in more detail in Supplementary Table 1. Stability was defined as the first epoch at which the derivative of the fitted curve changed sign from negative to positive, indicating the point beyond which additional data no longer meaningfully reduced variability. Analyses were conducted in STATA 18.

Using both approaches provides a more robust and interpretable estimate of stability than either method alone. The Statistical Convergence Approach offers a direct, threshold-based assessment of when interim estimates become empirically indistinguishable from the 100-epoch reference, whereas the Model-Based Inflection Approach captures the overall trajectory of variability reduction and identifies when additional data no longer meaningfully improves the estimate. Because statistical tests can be sensitive to transient fluctuations and polynomial models can smooth away abrupt changes, agreement between the two provides stronger evidence of true stabilization and reduces the risk of misclassification driven by noise or model form.

#### 2.5.3 Non-parametric evaluation of statistical convergence

We utilized bootstrap resampling and non-parametric permutation-based tests to quantify the underlying variance, establish formal confidence intervals, and rigorously test for systemic dependencies that influences the stabilization threshold. All procedures utilized 10,000 iterations to ensure stable null distributions. Across all tests and iterations, the stabilization threshold was iteratively recalculated using the identical Wilcoxon signed-rank criteria applied to the original observed data. Only iterations that converged within 100 epochs were included in the statistical evaluation using empirical *p-value*.

First, an Empirical Bootstrap Resampling of participants was used to obtain robust population-level median of stabilization threshold and 95% confidence intervals for the combined cohort and each clinical group. In each iteration, the sessions were sampled with replacement to match the number of each group (All groups, RTT, and TD), and the minimum stabilization threshold was computed. A two-tailed empirical *p-value* was obtained for RTT vs. TD group differences in minimum epochs based on the proportion of bootstrapped median differences crossing zero. The formula for the empirical *p-value* is noted below:


$$P_{EBR}=2\times min\left(\frac{1}{N}\sum_{i=1}^{N}I\left(boot,i\geq 0\right)\;,\;\frac{1}{N}\sum_{i=1}^{N}I\left(boot,i\leq 0\right)\right)$$

where _*boot, i*_ is the difference in medians (e.g., RTT-TD) for the *i*-th iteration. *I*(*condition*) represents an indicator, where 1 if the condition is true, and 0 if false.

Concurrently, the signal instability rate was computed, defined as the percentage of iterations that failed to reach convergence within 100 epochs. Group differences in this convergence failure rate were assessed using a Fisher's Exact Test.

Second, a Condition-Label Permutation test evaluated absolute differences in stabilization threshold between RTT and TD groups. The two group labels were randomly shuffled across the combined participant pool, and the stabilization threshold was computed to generate a null distribution of absolute RTT-TD differences. A two-tailed empirical *p-value* was derived by calculating the exact proportion of these permuted absolute differences that were greater than or equal to the observed absolute group difference. The formula for the empirical *p-value* is noted below:

$$\begin{array}{l} p_{CLP}=\frac{1}{N}\overset{N}{\underset{i=1}{\sum }}I\left({|}_{perm,i}|\geq_{obs}\right) \end{array}$$

where _*perm, i*_ is the difference between the randomly shuffled groups for the *i*-th iteration. _*obs*_ is the true empirical difference observed in the original data.

Third, a Temporal Scrambling analysis tested whether feature stability was driven by underlying temporal autocorrelation rather than strictly by cumulative data volume. The chronological sequence of artifact-free epochs was randomized independently within each participant's recording. A two-tailed empirical *p-value* was then derived by calculating the exact proportion of the stabilization thresholds from scrambled data that were greater than or equal to the observed minimum, alongside the proportion less than or equal to the observed minimum and doubling the smaller of the two values. The formula for the empirical *p-value* is noted below:

$$\begin{array}{l} p_{TS}=2\times min\\ \;\left(\frac{1}{N}\sum_{i=1}^{N}I\left(T_{scram,i}\geq T_{obs}\right),\;\frac{1}{N}\sum_{i=1}^{N}I\left(T_{scram,i}\leq T_{obs}\right)\right) \end{array}$$

where *T*_*scram, i*_ is the minimum stabilization threshold calculated for the *i*-th iteration, and *T*_*obs*_ is the actual minimum stabilization threshold observed in the chronological data.

Finally, an Age-Stratified Permutation test assessed developmental effects on data requirements. Participants were divided into early childhood (1 to 5 years, N_S_ = 60), middle childhood (6 to 10 years, N_S_ = 76), and adolescence (11 to 19 years, N_S_ = 59). To quantify variance across these stages, we calculated the sum of squared deviations and the maximum range of stabilization times across groups. Participant age labels were then randomly shuffled while preserving original group sample sizes. A one-tailed empirical *p-value* was derived for each metric based on the proportion of permuted dispersion values exceeding the empirically observed variance. A one-tailed evaluation was utilized because both the sum of squares and the maximum range are absolute, positive measures of dispersion. Consequently, we were exclusively testing the directional hypothesis that the true age stratification produces significantly greater between-group variance than random chance, making only the upper tail of the null distribution relevant for determining statistical significance. The formula for the empirical *p-value* is noted below:

$$\begin{array}{l} p_{ASP}=\frac{1}{N}\overset{N}{\underset{i=1}{\sum }}I\left(M_{perm,i}\geq M_{obs}\right) \end{array}$$

Where *M*_*perm, i*_is the dispersion metric (sum of squares or range) calculated for the *i*-th iteration, and *M*_*obs*_is the actual dispersion metric observed in the original age-stratified data.

#### 2.5.4 Sensitivity analysis of the stability reference window

To ensure our findings were not dependent upon an arbitrary choice of baseline, we conducted a formal sensitivity analysis to evaluate the impact of the reference window length. The stability reference windows for both the Statistical Convergence and Model-based Inflection approaches were reduced to 50 epochs (200 s) and 75 epochs (300 s). For the Statistical Convergence framework, empirical bootstrap resampling and condition-label permutation procedures (10,000 iterations) were recomputed at each reference length. This provided a rigorous verification of the robustness of the absolute minimum time estimates under scenarios of constrained data availability. Within the Model-Based Inflection framework, the model fitting procedure was repeated using the same reduced reference epochs. We assessed model performance by evaluating the consensus among six goodness-of-fit measures across the degrees of polynomial model ranging from first to fourth degree. This enabled a direct comparison of model reliability and reference stability across varying reference windows.

#### 2.5.5 RMBA correlation using minimum epochs

Prior work has demonstrated robust relationships between RTT clinical severity and spectral power (5). To assess whether shortened datasets preserved clinically meaningful associations between EEG features and behavior, partial correlations were computed between band power and Revised Motor Behavioral Assessment (RMBA) (18) scores while controlling for age. The correlation analysis used a subset of data comprising of 57 baseline EEG sessions from 48 RTT and 9 Suspected RTT recordings. For each frequency band, partial correlations were computed using band power computed at four data lengths: (1) the stabilization threshold defined by the Statistical Convergence Approach; (2) the stabilization threshold defined by the Model-Based Inflection Approach; (3) the first 100 epochs utilized as the stabilization reference; and (4) all available clean epochs for the given session. For each partial correlation coefficient, a 95% confidence interval (CI) was estimated via the Fisher z-transformation to approximate the sampling distribution of the correlation.

Furthermore, a *post-hoc* simulation was conducted to test a more ecologically valid acquisition scenario. Although the preceding analyses established the minimum volume of artifact-free data required for feature stability, this metric does not directly translate to a recommended raw recording duration. This distinction is necessary because preprocessing steps that rely on data-driven thresholds, such as Artifact Subspace Reconstruction (ASR), require the identification of clean calibration segments within the provided recording. Consequently, the feature outputs and stabilization thresholds obtained from 10 min of raw data might not necessarily translate to recordings with significantly reduced acquisition times. To validate the observed minimum in an ecologically valid context, the raw continuous EEG from the same 57 sessions were truncated before any processing steps. Factoring in the 95% data retention rate observed during pipeline validation, a benchmark of 3 min (180 s, or 45 epochs) of raw recording was selected. This specific duration was based on the most conservative observed stabilization threshold of 37 epochs (148 s), while simultaneously establishing a clean, practical time limit for realistic clinical EEG settings. The truncated data were then preprocessed using the correction-based pipeline, and the identical band power features were extracted. Lastly, a partial correlation with RMBA scores, controlling for age, was performed to determine whether this simulated real-world scenario of shorter recording successfully replicated the established clinical relationship.

## 3 Results

This work aimed to determine the minimum amount of EEG data needed to extract stable, reliable quantitative EEG (qEEG) features from clinical recordings. To maximize data retention, all data were processed through a standardized, fully automated correction-based pipeline, ensuring consistency, transparency, and scalability. After validating that the correction-based approach yielded features comparable to the rejection-based benchmark from prior works, we employed two complementary analytical methods to assess feature stability as a function of cumulative recording length. Specifically, we quantified the reduction in feature stability as additional 4-s epochs were added, identifying the point of empirical convergence against a long-duration reference, as well as modeling the overall trajectory of the data to locate the precise inflection point where additional recording time no longer meaningfully reduced variability.

### 3.1 Validation of the correction-based pipeline

To evaluate the performance of the proposed correction-based pipeline, the percentage of retained data and its distribution were compared with prior rejection-based benchmark and visualized (Figure 4). The correction-based pipeline retained significantly more data with a mean of 95.0% (median = 97.4%, *SE* = 0.9%, *IQR* = 4.7%), compared to a mean of 28.4% for the rejection-based benchmark (median = 27.0%, *SE* = 2.2%, *IQR* = 34.0%). A two-sample *t*-test confirmed that this difference in the percentage of retained data was highly significant [*t*_(132)_ = −27.66, *p* < 0.001]. In addition, the traditional rejection-based pipeline resulted in a loss of three RTT sessions and one TD session due to no data remaining after rejection.

Visualizing the distribution of retained data from correction-based pipeline revealed a primary cluster of high-yield sessions (Figure 5). Specifically, 85.9**%** of the processed sessions retained more than 100 clean 4-s epochs, with 98.2% retaining over 50 epochs (200 s) and 21.1% exceeding 150 epochs (600 s). This high absolute data yield not only demonstrates the practical efficacy of the pipeline but also establishes a robust mathematical foundation for subsequent analyses. Consequently, this distribution naturally supports the implementation of a 100-epoch inclusion threshold to effectively filter out low-quality outliers while preserving a heavily powered clinical sample. The result on the sensitivity analysis using different reference thresholds is reported in the later section.

**Figure 6:**
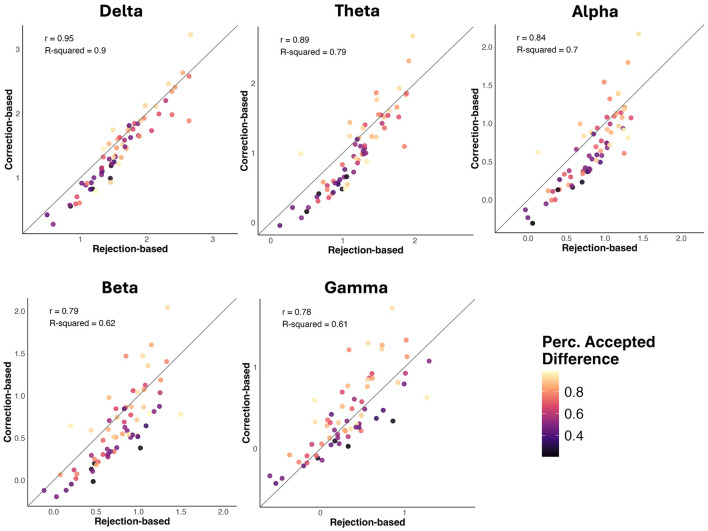
Scatterplots comparing log-transformed band power extracted from the rejection-based pipeline vs. the correction-based pipeline for each frequency band. Each point represents a participant, and colors indicate the difference in percentage of data retained between pipelines (correction-based minus rejection-based). The diagonal line denotes unity (x = y), where points falling along the line reflect identical feature estimates between pipelines. Across all bands, measurements derived from the correction-based pipeline closely matched those from the rejection-based pipeline, with corresponding correlation coefficients and *R*^2^ values shown in each panel.

Feature-level correspondence between pipelines was strong across all frequency bands, with correlation coefficients ranging from *r* = 0.78 to 0.95 (*R*^2^ = 0.61 to 0.90). As shown in Figure 6, power estimates from the correction-based pipeline closely matched those from the prior workflow, with points clustering tightly around the identity line. While recordings with larger discrepancies in data retention exhibited slightly greater divergence, overall agreement remained high across all frequency bands.

### 3.2 Minimum number of epochs to achieve stability

To determine the minimum amount of data required for stable spectral estimation from the correction-based pipeline output, we utilized both the Statistical Convergence and Model-Based Inflection approaches.

#### 3.2.1 Statistical convergence approach and group differences

**Figure 7:**
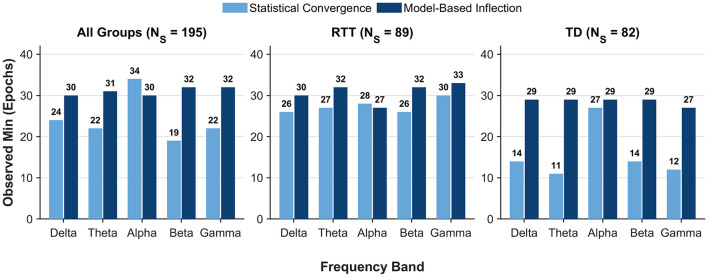
Comparison of observed minimum epochs required for stability across frequency bands. The bar plots display the observed minimum number of epochs required to achieve stability in feature estimates, utilizing a reference window of 100 epochs. Performance was compared between two complementary approaches: Statistical Convergence (light blue bars) and Model-Based Inflection (navy bars). The numbers above each bar denote the exact minimum number of epochs required for stability. Results are stratified by clinical cohort: All Groups combined (*N*_*S*_ = 195), Rett syndrome (RTT; *N*_*S*_ = 89), and Typically Developing (TD; *N*_*S*_ = 82). The minimum epochs required for stabilization were evaluated across five canonical EEG frequency bands (Delta, Theta, Alpha, Beta, and Gamma).

**Figure 8:**
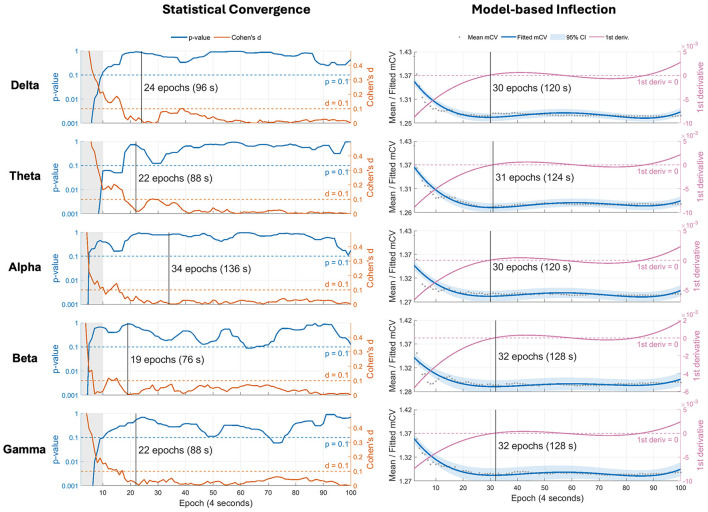
Per-frequency curves showing decision metrics for the two stability estimation approaches. The left panels show the Statistical Convergence approach, including *p-values* from Wilcoxon Signed Rank test and Cohen's *d*, with respective decision threshold in dotted horizontal line. The gray shaded region represents biological plausibility constraint, up to 10 epochs. The stabilization threshold indicating convergence estimate is marked in a vertical line with corresponding epoch count. The right panel shows the Model-based Inflection approach, with gray dotted curves representing the mean mCV, blue solid line representing fitted mCV with 95% confidence interval, and its first derivatives. The minimum epoch indicating inflection points is marked in a vertical line with corresponding epoch count. Each row represents the conventional frequency bands. Together, the panels demonstrate consistent frequency-specific patterns across methods, with the Model-based Inflection approach yielding slightly more conservative epoch estimates.

The Statistical Convergence approach identifies the threshold where cumulative variability estimates become empirically indistinguishable from a stable long-duration reference. By establishing a rigid statistical boundary, this method provides a discrete minimum data requirement for robust feature extraction. The minimum number of epochs for each frequency band and clinical group are summarized in Table 1 and Figures 7, 8. Within the observed empirical data for all combined groups and sessions (N_S_ = 195), stabilization occurred at 24 epochs for delta (96 s), 22 epochs for theta (88 s), 34 epochs for alpha (136 s), 19 epochs for beta (76 s), and 22 epochs for gamma (88 s). In addition, the RTT group (N_S_ = 89) resulted in stabilization at 26 epochs for delta (104 s), 27 epochs for theta (108 s), 28 epochs for alpha, (112 s), 26 epochs for beta (104 s), and 30 epochs for gamma (120 s). In the TD group, generally lower stabilization threshold was observed than in the RTT group-−14 epochs for delta (56 s), 11 epochs for theta (44 s), 27 epochs for alpha (108 s), 14 epochs for beta (56 s), and 12 epochs for gamma (48 s).

**Table 1:** Minimum epochs for feature stability derived via the statistical convergence approach and validated by non-parametric evaluation methods.

Band	Metric	All Sessions (*N_*S*_* = 195)	RTT (*N_*S*_* = 89)	TD (*N_*S*_*= 82)	RTT vs. TD
Delta	Observed minimum	24 (96 s)	26 (104 s)	14 (56 s)	*P*_*CLP*_ > 0.05
	Median bootstrap minimum	24 (96 s)	28 (112 s)	18 (72 s)	*P*_*EBR*_ > 0.05
	95% CI	11–66 (44–264 s)	12–96 (48–384 s)	10–47 (40–188 s)	-
	Instability rate (%)	0.08	2.32	1.00	*P*_*Fis*_ > 0.001
Theta	Observed minimum	22 (88 s)	27 (108 s)	11 (44 s)	*P*_*CLP*_ > 0.05
	Median bootstrap minimum	22 (88 s)	29 (116 s)	14 (56 s)	*P*_*EBR*_ > 0.05
	95% CI	12–44 (48–176 s)	10–97 (40–388 s)	10–93 (40–372 s)	-
	Instability rate (%)	0.06	8.34	1.94	*P*_*Fis*_ > 0.001
Alpha	Observed minimum	34 (136 s)	28 (112 s)	27 (108 s)	*P*_*CLP*_ > 0.05
	Median bootstrap minimum	22 (88 s)	24 (96 s)	18 (72 s)	*P*_*EBR*_ > 0.05
	95% CI	10–74 (40–296 s)	10–95 (40–380 s)	10–61 (40–244 s)	-
	Instability rate (%)	0.19	2.44	0.16	*P*_*Fis*_ > 0.001
Beta	Observed minimum	19 (76 s)	26 (104 s)	14 (56 s)	*P*_*CLP*_ > 0.05
	Median bootstrap minimum	20 (80 s)	26 (104 s)	15 (60 s)	*P*_*EBR*_ > 0.05
	95% CI	10–55 (40–220 s)	10–70 (40–280 s)	10–64 (40–256 s)	-
	Instability rate (%)	0.07	1.26	0.27	*P*_*Fis*_ > 0.001
Gamma	Observed minimum	22 (88 s)	30 (120 s)	12 (48 s)	*P*_*CLP*_ > 0.05
	Median bootstrap minimum	22 (88 s)	27 (108 s)	15 (60 s)	*P*_*EBR*_ > 0.05
	95% CI	11–63 (44–252 s)	11–88 (44–352 s)	10–66 (40–264 s)	-
	Instability rate (%)	0.38	1.96	2.87	*P*_*Fis*_ > 0.001

This table presents the observed minimum epochs, and the median bootstrapped minimums (10,000 iterations) required to achieve feature stabilization for the combined cohort (N_S_ = 195; including RTT, S-RTT, and TD), the Rett syndrome group (RTT; N_S_ = 89), and the Typically Developing group (TD; N_S_ = 82). The 95% confidence intervals (CI) reflect the distribution of minimum epochs among the successfully converged bootstrap iterations. The Instability Rate (%) denotes the percentage of bootstrap iterations that failed to reach a stable minimum within the available data limits. Group differences in stabilization time between the RTT and TD cohorts were evaluated using two complementary methodologies: a Condition-Label Permutation test (*p*_*CLP*_) to evaluate the observed minimum, and an Empirical Bootstrap Resampling analysis (*p*_*EBR*_) to evaluate the median bootstrap minimums. Neither test showed a statistically significant RTT vs. TD difference in the minimum epoch requirements across any frequency band. Conversely, a Fisher's Exact Test revealed a highly significant group difference in the instability rate, demonstrating a much higher prevalence of non-convergence iterations in the RTT group across all frequency bands (*p*_*Fis*_ < 0.001).

#### 3.2.2 Permutation-based evaluation of statistical convergence

Following the determination of the convergence thresholds, four permutation-based analyses with 10,000 iterations were conducted to quantify variance and test specific clinical and demographic hypotheses.

First, an Empirical Bootstrap Resampling was utilized to derive robust population-level estimates. The median bootstrap minimums closely mirrored the observed data, yielding 24 epochs [96 s; 95% CI = (11–66) epochs] for delta, 22 [88 s; 95% CI = (11–66) epochs] for theta, 22 [88 s; 95% CI = (10–74) epochs] for alpha, 20 [80 s; 95% CI = (10–55) epochs] for beta, and 22 [88 s; 95% CI = (11–63) epochs] for gamma (Table 1). In addition, this resampling procedure facilitated a bootstrap difference analysis and explicitly quantified the signal instability rate. The bootstrap difference analysis confirmed that the absolute minimum epoch requirements did not significantly differ between the RTT and TD cohorts across any frequency band (*p*_*EBR*_>0.05; Table 1). Conversely, a Fisher's Exact Test applied to the bootstrap iterations revealed a highly significant clinical dichotomy. RTT recordings exhibited a significantly higher instability rate compared to TD recordings across all spectral bands, demonstrating a greater propensity for non-convergence within the available data limits (*p*_*Fis*_ < 0.001; Table 1).

Second, a Condition-Label Permutation test evaluated statistical differences in stabilization rates between the clinical cohorts. While the RTT group nominally required more epochs to stabilize than the TD group across all frequency bands, the permutation analysis revealed that these differences in absolute minimum epoch requirements were not statistically significant (*p*_*CLP*_ > 0.05).

Third, a Temporal Scrambling analysis was applied to explicitly test whether feature stability was influenced by underlying sequential structure of the data. Comparing the convergence thresholds derived from the scrambled data to those from the chronological data revealed no significant temporal dependency in any condition or age group (*p*_*TS*_ > 0.05). This confirms that feature stability is strictly a function of cumulative artifact-free data volume rather than the chronological sequence of the epochs.

Finally, an Age-Stratified Permutation test assessed developmental effects on data requirements. Participants were stratified into three discrete age groups (1 to 5 years, 6 to 10 years, and 11 to 18 years). The permutation-based age dependency tests revealed no statistically significant effects using either the Sum of Squares or range metrics across any spectral band (*p*_*ASP*_ > 0.05). In sum, the rate of feature convergence does not significantly depend on patient age, indicating that the established minimum time thresholds remain robust and applicable across the entire pediatric span investigated in this study.

#### 3.2.3 Model-based inflection approach

The Model-Based Inflection Approach maps the overarching trajectory of variability reduction across time to locate the exact inflection point where the additional data ceases to provide meaningful improvements, indicated by change in the sign of derivative from negative to positive. This mathematical framework smooths out transient fluctuations to yield a conservative estimate of overarching signal stabilization. Within this framework, all combined groups and sessions (*N*_*S*_ = 195) showed stability at 30 epochs for delta (120 s), 31 for theta (124 s), 30 for alpha (120 s), 32 for beta (128 s), and 32 for gamma (128 s). RTT group (*N*_*S*_ = 89) resulted in stabilization at 30 epochs for delta (120 s), 32 epochs for theta (108 s), 27 epochs for alpha (108 s), 32 epochs for beta (128 s), and 33 epochs for gamma (132 s). Similar to the Statistical Convergence approach, the TD group showed generally lower stabilization threshold than the RTT group-−29 epochs for delta, theta, alpha, and beta (116 s), and 27 epochs for gamma (108 s; Figures 7, 8). Both analytical approaches demonstrated consistent frequency-specific patterns across participant cohort, reinforcing the reliability of the established minimum data requirements.

### 3.3 Sensitivity analysis of the stable reference epochs

**Figure 9:**
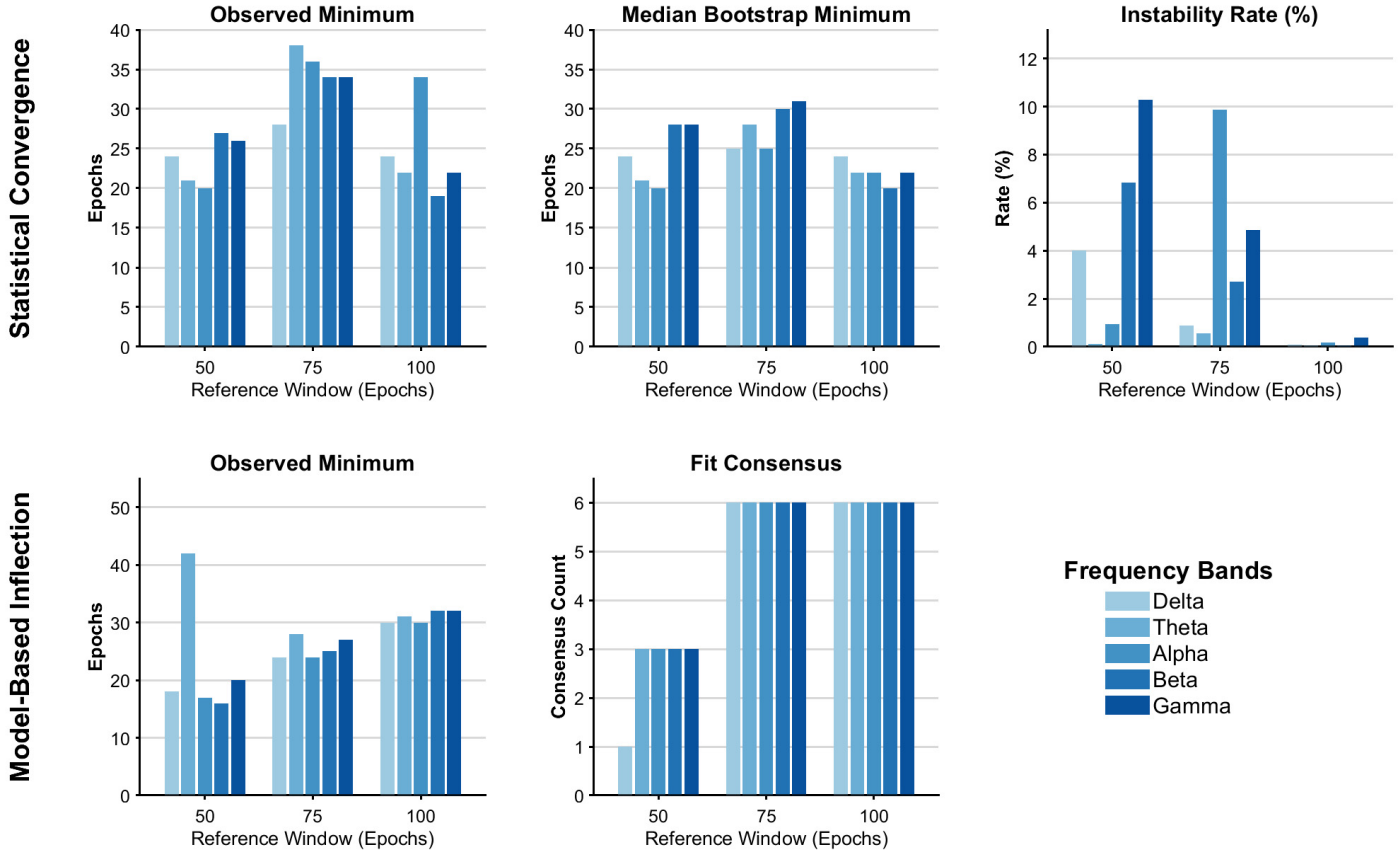
Sensitivity analysis of the stability reference window duration. The grouped bar charts illustrate the impact of varying the reference window length (50, 75, and 100 epochs) on the reliability of the minimum time estimates across the five frequency bands. The top row displays metrics for the Statistical Convergence approach, including the observed minimum epoch count, the median bootstrap minimum, and the instability rate (percentage of failed iterations). The bottom row displays metrics for the Model-Based Inflection approach, specifically the observed minimum epoch count and the consensus count of the six goodness-of-fit criteria. Within each panel, the sequential blue color gradient represents the frequency bands from Delta (lightest blue) to Gamma (darkest blue). Note that the 50 and 75-epoch reference windows resulted in significantly higher instability rates and lower model consensus, whereas the 100-epoch reference provided robust and stable estimates across all measures and frequency bands.

Sensitivity analyses demonstrated that reducing the reference length to 50 or 75 epochs compromised the robustness of the stabilization estimates (Figure 9). Within the Statistical Convergence framework, these shorter reference windows resulted in elevated instability rates. Specifically, the instability rate reached 10.28% in the gamma band for the 50-epoch condition and 9.88% in the alpha band for the 75-epoch condition. The Model-Based Inflection approach exhibited similar vulnerability at the 50-epoch threshold, where the fourth-degree polynomial model failed to achieve consensus across the goodness-of-fit criteria. Although the 75-epoch reference showed an improved fit consensus, it still yielded unacceptably high instability within the convergence framework. Ultimately, utilizing a 100-epoch reference eliminated these methodological vulnerabilities in both the Statistical Convergence and Model-Based Inflection approaches. This longer window ensured near-zero instability and high model fidelity across all frequency bands. These results confirm that a 100-epoch window provides the most reliable benchmark to serve as a stable reference estimate.

### 3.4 RMBA correlation using minimum amount of epochs

Across all frequency bands, partial correlations between spectral power and RMBA scores remained highly consistent when using only the minimum number of epochs identified by the two proposed methods compared with the Fixed Reference or Full Recording (Table 2). Correlation coefficients using the Statistical Convergence and Model-Based Inflection thresholds differed by a magnitude of 0.05 or less from those obtained using the total available data. Importantly, the 95% confidence intervals for these correlations overlapped across all conditions, indicating that the clinical effect sizes remained stable even when data volume was reduced. All associations remained statistically significant after controlling for age, which suggests that the identified minimum recording durations are sufficient to capture the underlying relationship between neurophysiological biomarkers and clinical measures.

**Table 2:** Correlation coefficient (*r*), *p-value*, and 95% confidence interval of RMBA partial correlation with band power after controlling for age.

Frequency	Statistical convergence	Model-based inflection	Fixed reference (100 epochs)	Full recording	3-Minute Reprocessed
Delta	*r* = 0.504 *p* < 0.001 CI = [0.28–0.68]	*r* = 0.503 *p* < 0.001 CI = [0.28–0.68]	*r* = 0.499 *p* < 0.001 CI = [0.27–0.67]	*r* = 0.517 *p* < 0.001 CI = [0.29–0.69]	*r* = 0.483 *p* < 0.001 CI = [0.25–0.66]
Theta	*r* = 0.436 *p* = 0.001 CI = [0.20–0.63]	*r* = 0.431 *p* = 0.001 CI = [0.19–0.62]	*r* = 0.421 *p* = 0.001 CI = [0.18–0.62]	*r* = 0.474 *p* < 0.001 CI = [0.24–0.66]	*r* = 0.406 *p* = 0.002 CI = [0.16–0.60]
Alpha	*r* = 0.410 *p* = 0.002 CI = [0.19–0.62]	*r* = 0.404 *p* = 0.002 CI = [0.16–0.60]	*r* = 0.404 *p* = 0.002 CI = [0.16–0.60]	*r* = 0.453 *p* < 0.001 CI = [0.22–0.64]	*r* = 0.392 *p* = 0.003 CI = [0.14–0.59]
Beta	*r* = 0.427 *p* = 0.001 CI = [0.19–0.62]	*r* = 0.430 *p* = 0.001 CI = [0.19–0.62]	*r* = 0.424 *p* = 0.001 CI = [0.18–0.62]	*r* = 0.456 *p* < 0.001 CI = [0.22–0.64]	*r* = 0.394 *p* = 0.003 CI = [0.15–0.60]
Gamma	*r* = 0.415 *p* = 0.001 CI = [0.17–0.61]	*r* = 0.407 *p* = 0.002 CI = [0.16–0.61]	*r* = 0.408 *p* = 0.002 CI = [0.16–0.61]	*r* = 0.428 *p* = 0.001 CI = [0.19–0.62]	*r* = 0.371 *p* = 0.005 CI = [0.12–0.58]

The features were computed by averaging the 4-s windows at different minimum epochs determined using Statistical Convergence, Model-based Inflection, Fixed Reference (100 epochs), and Total Available Epochs. Lastly, the truncated data at raw data step into 3 min were reprocessed and the extracted features were tested. The minimum stabilization threshold from Statistical Convergence and Model-Based Inflection, as well as the 3-Minute Reprocessed, replicate the significant relationship with RMBA scores.

### 3.5 Ecological validation of the minimum epoch

When the raw EEG recordings were truncated to 3 min and reprocessed through the entire correction-based pipeline, the data retention rate remained high, averaging 93.0 % (SD = 6.0%). Furthermore, the spectral features extracted from these truncated recordings preserved clinical utility. As shown in “3-Minute Reprocessed” column in Table 2, the resulting band power estimates demonstrate statistically significant partial correlations with the RMBA scores across all evaluated frequency bands (Delta: *r* = 0.483, *p* < 0.001; Theta: *r* = 0.406, *p* = 0.002; Alpha: *r* = 0.392, *p* = 0.003; Beta: *r* = 0.394, *p* = 0.003; Gamma: *r* = 0.371, *p* = 0.005). These findings confirm that the correction-based pipeline performs reliably even when constrained to a 3-min raw acquisition window.

## 4 Discussion

In this study, we demonstrated that a fully automated, correction-based EEG preprocessing pipeline can substantially improve data retention and consistency in a large, multisite Rett syndrome dataset while producing spectral features that closely match those from a rejection-based workflow used in prior studies. Using data processed with the correction-based pipeline, we applied two complementary approaches—a non-parametric statistical convergence test and a model-based inflection analysis—to quantify how spectral variability decreased as additional clean 4-s epochs were accumulated. Both methods resulted in a minimum of approximately 19–37 epochs (76–148 s) as the amount of data required for stable spectral power estimates across frequency bands. Importantly, features computed from these shortened datasets preserved strong and significant associations with clinical severity.

### 4.1 Rejection-based vs. correction-based pipeline

The correction-based preprocessing pipeline substantially improved data retention while producing spectral features that closely matched those obtained from the prior rejection-based workflow. The correction-based pipeline retained a substantially greater proportion of data (mean of 95%) than the rejection-based workflow (mean of 28%), while maintaining strong correspondence in spectral features across all frequency bands. The rejection-based approach took a strict “data purist” approach, where minimal processing on the data is performed as an attempt to retain as much brain-sourced signal as possible. With the level of artifact in EEG recordings from RTT being high, a very conservative amplitude threshold (150 μV) had to be used, resulting in rejection of large portion of data. In the tradeoff between data retention and absolute signal purity, the previous works on RTT chose strict purity. On the other hand, correction-based pipeline utilizes more recent signal processing and correction techniques that statistically isolate and remove artifacts at different levels of stationarity, enabling higher retention of data length at the expense of potentially losing brain-sourced data that might not be completely separated in component decomposition step. However, the current optimized pipeline was able to show a good correspondence to the prior rejection-based pipeline and replicate significant correlation with clinical measures.

### 4.2 Validation of correction-based pipeline

The automated processing steps in the correction-based pipeline include: (1) Bad channel rejection using Local Outlier Factor, (2) Artifact Subspace Reconstruction (ASR) followed by window rejection, and (3) Automatic ICA component rejection using ICLabel. This implementation addresses a central limitation in pediatric and neurodevelopmental EEG research, where manual or amplitude-based segment rejection often results in large data losses and inconsistent operator-dependent decisions. Higher data retention not only improves analytic reliability but also has direct clinical and practical significance: when more of the recorded EEG is usable, shorter acquisition times can yield sufficient clean data, reducing the overall burden for participants. This is especially important in Rett syndrome, where tolerance for long sessions may be limited by motor dysfunction, discomfort, limitations in communication, or behavioral challenges (7). Together, this work demonstrates that the correction-based pipeline can enhance data quantity and consistency while supporting more efficient, participant-friendly EEG protocols in clinical studies. In the following sections, we discuss the implications and practical concerns of using the proposed correction-based pipeline.

#### 4.2.1 Channel rejection using local outlier factor (LOF)

Local Outlier Factor (LOF) algorithm identifies channels exhibiting abnormal spatial–temporal patterns that are common in high-artifact pediatric EEG. In this implementation, LOF was paired with an adaptive rejection threshold, allowing the algorithm to distinguish between true artifacts and clinically meaningful variability that often presents as large-amplitude or low-frequency fluctuations in RTT EEG. This adaptivity is particularly important in neurodevelopmental datasets, where atypical but physiologically relevant signal characteristics may otherwise be removed by more rigid channel-rejection rules. Consistent with this rationale, LOF-based rejection showed closer agreement with the manual channel selection than a RANSAC-based approach (see Supplementary Figure 1), which may help explain the strong replication of spectral features between the correction-based and rejection-based pipelines. By incorporating adaptive LOF-based channel assessment, the present pipeline provides a principled approach for retaining clinically meaningful variation while mitigating artifact burden in multisite neurodevelopmental recordings.

#### 4.2.2 Segment correction using artifact subspace reconstruction (ASR)

Another crucial automated processing step in the correction-based pipeline was Artifact Subspace Reconstruction (ASR), followed by automatic window rejection, which served to clean non-stationary artifacts from the data. This sliding-window-based process of rejecting the artifactual component subspace and projecting the non-artifactual subspace back has been reported to be effective in different EEG experimental designs and recording setups (25–27). Our work is the first to report the use of ASR in the processing of EEG recorded from RTT patients. The approach successfully retained high volume of data while replicating the spectral features and clinical correlations observed in past studies. However, because ASR relies on identifying a “clean” portion of the recording to be used as a reference within the data itself, there is a practical concern regarding whether a sufficient clean reference can be found in recording of drastically reduced lengths. We validated this concern by simulating the ecologically valid scenario, cropping the raw continuous EEG data to 3 min and re-processing the pipeline so that ASR will be implemented with a reduced amount of data to find a “clean” reference. We still observed a strong correlation of qEEG features with RMBA scores.

#### 4.2.3 Independent component rejection using ICLabel

The final automated step in the preprocessing pipeline involves Independent Component Analysis (ICA) and ICLabel to effectively remove stationary artifacts such as ocular and muscular artifacts (31). A known limitation of the ICLabel classifier is that its training data consisted of mostly adult EEG recordings. Because infant and pediatric brain differs substantially in spatial and temporal dynamics, it is reported that the classifier might misclassify pediatric “brain” components into the “other” category (28). While this structural limitation impacts the classification of cortical sources, the current study utilizes ICLabel exclusively for the identification and removal of ocular and muscular artifacts. Because these non-neural artifactual signatures are robust and stereotypic across ages, adult-trained classifiers can still be a viable method to characterize ocular and muscular ICs in pediatric EEG. While there are pediatric-focused alternatives such as adjusted-ADJUST (32) and iMARA (33), they present their own limitations in the context of RTT EEG research. ADJUST-based algorithms are primarily targeted toward ocular artifacts, making it less suitable for RTT datasets characterized by high level of movement artifacts. While iMARA is a viable alternative, its performance relative to ICLabel remains undocumented in this specific clinical context. A compelling future alternative could be wavelet-based ICA (wICA)-ICA-MARA sequence used in HAPPE pipeline (16). Although validated on infant data, direct comparison against ICLabel is currently lacking in the literature, leaving the evaluation of this approach outside the scope of the present work.

### 4.3 Minimum data requirements for stable feature estimation

Determining the minimum amount of EEG data required for reliable feature estimation is essential for defining standardized acquisition protocols in clinical research. Both analytical approaches—the Statistical Convergence and Model-Based Inflection methods—yielded consistent estimates of feature stability across frequency bands. Across all participants, the minimum number of 4-s epochs required for stable spectral power ranged from 19–34 epochs (76–136 s) across all frequency bands and analytical approaches. In the Statistical Convergence approach, the minimum of 19 epochs was found in beta band while the maximum of 34 was found in the alpha band. This higher stabilization threshold in the alpha band appears to be driven by the strictness of the 15-epoch lookahead criterion rather than a distinct physiological stabilization trajectory. The bootstrapping analysis demonstrated that the median bootstrap minimum for alpha band to be on par with other frequency bands. Furthermore, visual inspection confirms that alpha power satisfied the primary stability thresholds (*p* > 0.1, Cohen's *d* < 0.1) as early as 19–24 epochs. This is consistent with other frequencies, but transient fluctuations occurring within the subsequent lookahead window delayed the formal declaration of stability. Model-Based Inflection approach resulted in more consistent estimate of minimum epoch count across frequencies compared to Statistical Convergence approach, with a total range of 2 epochs across frequency bands. However, the inflection measure resulted in overall higher epoch count than convergence approach, potentially providing more conservative stabilization requirement.

In sum, these result support that approximately 2.5 min of artifact-free resting-state EEG are sufficient to obtain stable spectral estimates across all conventional frequency bands. Using the fully automated, correction-based preprocessing pipeline with a mean of 95% data retention rate, the duration of raw EEG data collection can be extrapolated to be as little as 2.5–3 min—representing a substantial reduction from the ~10-min protocols we have used in prior (5, 7, 19) and on-going (R61- NCT05932589) work. Shorter acquisition times have direct implications for study design, enabling improved data yield, participant comfort, and feasibility of repeated assessments, particularly for individuals with limited tolerance for prolonged EEG procedures.

#### 4.3.1 Analysis of systemic dependencies in statistical convergence method

Although previous studies have suggested that lower-frequency bands require longer recordings to reach stability (9, 10), we did not observe a clear or systematic frequency-dependent pattern in our dataset, likely reflecting differences in population characteristics, preprocessing methods, or analytic approach. In addition to frequency bands, this work rigorously tested the systematic dependencies of minimum amount of data required for stability in clinical condition using the four approaches: (1) an Empirical Bootstrap Resampling, (2) Condition-Label Permutation, (3) Temporal Scrambling, (4) Age-Stratified Permutation tests to test for the effect of clinical condition, temporal order, and age group affects the minimum epoch result.

Empirical Bootstrap Resampling analysis and Condition-Label Permutation enabled group comparison of minimum number of epochs across RTT and TD. Both methods serve as complementary approaches where Empirical Bootstrap Resampling simulates population level differences and Condition-Label Permutation compares observed RTT-TD difference against a null distribution. Both approaches yielded no significant difference in minimum number of epochs. This is advantageous in the perspective of pipeline processing and recording length recommendation as the result supports the use of one universal recording length processed via the same correction-based pipeline on both clinical and healthy population.

Yet we observed one condition-specific difference in the instability rate in the Empirical Bootstrap Resampling analysis. Although both minimum number of epochs and instability rate represent the measure of variability based on the bootstrapped samples, the two measures reflect slight difference in nuance on how the variability is perceived. The group difference in the minimum number of epochs was tested using an empirical *p-value* approach, and only iterations that converged were taken into this measure. This represents a sample from the population where the quality and within-recording variability of measured EEG and the output from correction-based pipeline fall within the acceptable range. However, the instability rate which takes the proportion of non-convergent bootstrap iterations, simulates a more extreme case of noisy subsamples within the population, reflecting the inherent condition-specific difference where more extreme variability could be introduced. This lines up with previous findings of qEEG features from RTT displaying higher variability (5).

The other evaluations including Temporal Scrambling and Age-Stratified Permutation failed to show significant effect on minimum stabilization threshold. Temporal Scrambling test suggests that the observed minimum stabilization threshold is not due to particular temporal sequence and is independent of temporal autocorrelation but more sensitive to the cumulative number of epochs. Age-Stratified Permutation test also indicates that the minimum stabilization is independent of age groups, demonstrating the robustness of the current minimum stabilization threshold across developmental stages.

### 4.4 Evaluation of stability determination methods

Two complementary frameworks were used to determine the minimum number of epochs required for stable EEG feature estimation: the Statistical Convergence approach and the Model-Based Inflection approach. The Statistical Convergence approach defines stability relative to a fixed reference of 100 epochs (400 s), identifying the point at which interim variability is no longer statistically distinguishable from this benchmark. Although this approach is intuitive and easy to interpret, it depends on the assumption that the number of epochs used as a benchmark constitute a sufficiently stable reference. Furthermore, it assumes that the chosen *p-value* and Cohen's d cutoffs are representative of stability across different datasets or frequency bands. The Model-Based Inflection approach is fully data-driven and does not require the selection of variable cutoffs. By modeling the relationship between mCV and cumulative epoch count, it identifies the point at which additional data yield diminishing reductions in variability. However, this method still relies on a total data volume that is sufficient for capturing the underlying form of the stabilization plateau.

As the sensitivity analysis revealed, a broad temporal window is required to ensure robustness of the model and result in both frameworks. When the reference window is truncated to 50 or 75 epochs, the Statistical Convergence approach suffers from a sharp increase in instability rates. Simultaneously, the Inflection approach undergoes a model selection collapse because the truncated data provide insufficient curvature for the goodness-of-fit criteria to accurately identify a 4th-degree polynomial. In these shortened scenarios, the criteria erroneously favor simpler models that cannot capture the transition to stability. The selection of the 100-epoch window was informed by existing literature and the empirical distribution of clean data following our automated preprocessing pipeline. As illustrated in Figure 5, this duration encompasses the majority of available clean epochs across the cohort, ensuring that the stabilization estimates represent a true physiological plateau rather than a transient local state.

Across both methods, the stability estimates obtained (76–136 s) slightly exceed the minimums suggested in previous literature. Early adult studies reported that 40 to 60 s of artifact-free data were required to minimize variability (9, 10), while more recent large-scale adult analyses have refined this range to approximately 30 to 120 s depending on the frequency band (11, 12). In pediatric research, standards have been notably more heterogeneous; a recent expert survey identified a mean acceptable cutoff of 52 s for clean data, though reported thresholds ranged widely from 1 s to over 3 min (13). Our results suggest that obtaining reliable spectral features in Rett syndrome requires data durations between 1.5 and 2.5 min. This requirement is higher than the average pediatric “minimum” and likely reflects the specific challenges of atypical spectral structure and higher artifact burden in this population.

It is important to note that no prior work has directly examined feature stability in Rett syndrome or related genetic neurodevelopmental disorders, despite similar challenges regarding participant tolerance and data quality. The present findings extend the existing literature by offering the first data-driven estimates of spectral stability in RTT using fully automated and correction-based preprocessing pipeline. Demonstrating that approximately 76–136 s of artifact-free data are sufficient, places RTT research within the broader context of EEG sufficiency while underscoring the need for disorder-specific, methodologically standardized approaches for defining minimum data requirements.

### 4.5 Preservation of clinical signal–behavior associations

The spectral features derived from the minimum required epochs retained their meaningful associations with clinical severity. Across all frequency bands, partial correlations between band power and RMBA scores remained highly significant and nearly identical to those obtained from 100 epochs or the full dataset. The observed differences in correlation coefficients were minimal, with a maximum variance of 0.05 or less across all conditions. These findings demonstrate that when data length is guided by quantitative stability criteria, reducing the amount of analyzed EEG does not compromise the sensitivity of qEEG biomarkers to clinical outcomes. The robustness of delta power correlations with RMBA aligns with earlier reports linking elevated low-frequency power to disease severity and cortical dysfunction in RTT (5). Together, these results validate the stability-based framework as both methodologically sound and practically feasible for extracting clinically interpretable neural features in neurodevelopmental disorders.

The success of the simulated 3-min acquisition further illustrates the robustness of the automated preprocessing pipeline. The high data retention rate of 93.0 % on average suggests that shorter acquisition windows do not negatively impact the efficacy of artifact rejection of data-driven steps such as Artifact Subspace Reconstruction (ASR) which identifies stable calibration segments within the data. The high level of retention during the early portion of recording could be attributed to the cumulative impact of participant fatigue and movement-related artifacts, which are common challenges in longer clinical protocols. By preserving the signal-to-noise ratio within a shorter, beginning portion of the data, the automated pipeline ensures that the resulting features remain representative of the individual's underlying neurophysiology.

In summary, reliable brain–behavior associations can be obtained with substantially shorter recording durations when automated preprocessing and stability-based data selection are applied, supporting the feasibility of shorter yet scientifically rigorous EEG acquisitions in clinical and translational research.

### 4.6 Limitations

While this study establishes a robust framework for determining minimum EEG data requirements, the findings should be interpreted in the context of several methodological boundaries. First, the rejection-based preprocessing pipeline was applied only to a validation subset of the cohort due to practical time constraints; extending both pipelines to all recordings would provide a more comprehensive benchmark. Second, although the correction-based pipeline parameters were optimized using data-driven criteria including the delta–RMBA association, these tuning decisions may be dataset-specific and alternative configurations may be required in other NDD populations or acquisition environments. Third, while many participants contributed multiple EEG sessions, multiple within-subject recordings were analyzed as independent observations in this work for simplicity in stability determination. Fourth, requiring ≥100 clean epochs for inclusion may bias the analytic sample toward participants with milder motor symptoms or lower artifact burden, potentially underestimating stability requirements for individuals with more severe clinical features. Fifth, regarding methodological validation, while algorithms such as Artifact Subspace Reconstruction (ASR) and Independent Component Analysis (ICA) are well validated in typically developing populations, they have not been explicitly validated using simulated neural data reflecting the unique spatial and temporal artifact profiles of the RTT population. Sixth, the pipeline comparison used in this work must be interpreted cautiously due to the use of different filter designs, particularly regarding the delta and gamma frequency cutoffs. Because these boundary bands are differentially affected by filter boundaries, spectral slope interactions, log transformation, and periodogram estimation effects, the apparent agreement in spectral features may partly reflect these non-neurophysiological preprocessing factors rather than true equivalence in underlying spectral content. Furthermore, given the substantial heterogeneity in preprocessing approaches across the broader literature, absolute delta and gamma estimates may not be directly comparable across different studies or legacy pipelines. Lastly, this study examined stability only for spectral power; other qEEG measures (e.g., aperiodic parameters, connectivity, entropy) may have different data requirements, and developmental effects on stability thresholds were not explicitly evaluated.

### 4.7 Clinical and methodological implications

The combination of automated preprocessing and stability-guided data sufficiency analysis provides a reproducible framework for large-scale, multisite EEG research. Given the high percentage of data retained using the correction-based pipeline, these findings indicate that EEG recording sessions can be substantially shortened—approximately 3 min of clean data (45 epochs of 4 s) are sufficient for stable spectral estimates. This reduction has meaningful clinical impact: shorter sessions may improve data yield, participant comfort, and the feasibility of repeated assessments, particularly for individuals with limited tolerance for prolonged procedures.

Methodologically, automated preprocessing reduces manual workload, inter-rater variability, and data exclusion bias, which are key challenges faced by multicenter studies. By establishing data-driven thresholds for minimum recording duration, researchers can optimize session design to balance participant burden with analytic rigor. However, researchers must remain cautious when bridging legacy preprocessing methods with modern automated pipelines and implementing shorter time duration for stable feature estimates. Data-driven validation should come before creating potential change in the current recording and analysis protocol, as features of interest outside the scope of this study may require longer data, and processing steps specific to the study design may alter the data retention and feature variance in the dataset. The analytic framework introduced in this work can be useful in the determination of appropriate recording duration and is applicable beyond RTT to other pediatric and neurodevelopmental populations where movement artifacts and limited tolerance frequently constrain data quality.

### 4.8 Future directions

Future work should focus on comprehensive methodological validation and expansion. While automated cleaning algorithms including Artifact Subspace Reconstruction (ASR) and ICA-based rejections are well validated in typically developing populations, future studies should employ simulated neural data to explicitly validate these tools against the unique spatial and temporal artifact profiles of Rett syndrome. This step can be especially important when expanding the use of the proposed correction-based pipeline for additional analysis paradigms such as Event-Related Potentials (ERPs) or other evoked potentials. Additionally, as mentioned above, future research must examine whether the minimum stability thresholds differ for other quantitative EEG measures, such as aperiodic parameters, connectivity, and entropy, and determine if our data driven parameter tuning (e.g., the delta RMBA association) requires alternative configurations for other neurodevelopmental disorders. Additional research is needed to evaluate the temporal reliability of these stability thresholds and determine whether they vary with disease severity, medication status, or other clinical factors.

From the perspective of practical application, future work could extend these findings to real-time feature stability monitoring, allowing technicians to receive feedback during acquisition when sufficient clean data have been obtained. Integration of this framework with real-time artifact detection and adaptive data collection could further streamline EEG acquisition and improve data quality. In the longer term, as consumer-grade EEG systems increasingly demonstrate the ability to yield scientifically meaningful data in controlled contexts (34, 35), shorter recording protocols combined with advances in automated preprocessing and stability assessment may support more flexible acquisition paradigms, including higher-frequency longitudinal sampling outside traditional laboratory or clinical settings.

Furthermore, the bootstrapping analysis showed that RTT cohorts exhibited significantly higher instability. This finding raises a critical research question: does this elevated variance arise from intrinsic neural non-stationarity, potentially reflecting network dysregulation, or is it driven by residual artifacts unique to the RTT motor phenotype? Differentiating between neural volatility and artifactual noise will be essential for determining if stabilization length can serve as a novel biomarker. Ultimately, these developments will support scalable, reproducible EEG biomarker pipelines for clinical trials and natural history studies, accelerating the translation of quantitative EEG into practical outcome measures for RTT and related disorders.

## On behalf of (R61 EEG Biomarker Project Members)

Children's Hospital of Philadelphia: Holly Dubbs, Erin Prange, Dennis Fleysh, and Caroline Kessler; Children's Hospital of Los Angeles: Drs. Shafali Jeste and Payal Gu, and Sydney Jacobs; Children's Hospital Colorado: Dr Tim Benke and Lauren Mitchell; Vanderbilt University Medical Center: Drs Jeff Neul and Cary Fu, Nicole Thompson and Dorita Jones; Boston Children's Hospital: Drs. David Leiberman and April Levin, and Karen Sabol; Texas Children's Hospital: Dr. Bernhard Suter, Safaa Daou, and Walter Williamson.

## Data Availability

The data analyzed in this study is subject to the following licenses/restrictions: The data that support the findings of this study are not openly available but are available upon reasonable request from the corresponding author subject to IRB and governance approvals. Requests to access these datasets should be directed to Eric D. Marsh, marshe@chop.edu.
