# Supplementary material for: Minimum data requirements and automated preprocessing for reliable EEG biomarkers in Rett syndrome
Source: Front Neurol. 2026 Jun 16;17:1791834. doi: 10.3389/fneur.2026.1791834 (PMC13314485; doi:10.3389/fneur.2026.1791834)
Supplement: Supplementary file 1 [file Supplementary_file_1.docx]

Supplementary Material

# Supplementary Methods and Results

## Pipeline Optimization

The automated pipeline was optimized in two main stages: (1) evaluation of automated bad-channel detection methods, and (2) parameter tuning of the ASR algorithm.

### Automated Bad-channel Rejection Methods

Two complementary algorithms were compared: the Clean_rawdata plugin, which identifies channels based on RANSAC method (Bigdely-Shamlo et al., 2015), and the Local Outlier Factor (LOF) approach in NEAR (Kumaravel et al., 2022), which detects spatial-temporal outliers via density-based clustering. Performance was quantified using the F1 score, defined as

$$F1 score= 2\times\frac{Precision\times Recall}{Precision+Recall}=\frac{2\times TP}{2\times TP+FP+FN}$$

which balances precision and recall without bias from true negatives—a property suitable for sparse outlier detection in EEG.

As shown in Supplementary Figure 1, both methods correctly identified a subset of noisy electrodes (true positive; green cells). Although CR resulted in higher number of true positives, the LOF approach demonstrated superior overall accuracy and balance between sensitivity and precision. The clean_rawdata algorithm yielded an F1 score of 0.23, reflecting a tendency toward under-detection (high false-negative rate), whereas the LOF method achieved an F1 score of 0.42, indicating substantially improved detection of true noisy channels with fewer misclassifications. The higher F1 score for LOF suggests that the density-based outlier framework better captures spatially localized noise patterns that are common in EEG in severe neurodevelopmental disorders including RTT, supporting its selection as the default bad-channel rejection method in the automated preprocessing pipeline.

To evaluate the relative performance of automated bad-channel detection methods, we compared the LOF algorithm with the RANSAC-based implementation in clean_rawdata. LOF identified spatially deviant channels more consistently in RTT datasets that contained localized motion or impedance-related artifacts. RANSAC, which relies on correlation with reconstructed reference channels, tended to under-detect channels with non-stationary noise. These patterns are consistent with previous reports (Kumaravel et al., 2022).

### Artifact Subspace Reconstruction (ASR) parameters

ASR parameters were systematically varied to optimize artifact removal and signal preservation. Burst criteria (5–25) and sliding-window lengths (0.5–8 s) were explored. The burst criterion defines the standard deviation threshold of the clean EEG baseline above which transient high-variance activity is classified as an artifact burst and reconstructed, whereas the sliding-window length sets the temporal scale over which covariance and variance are estimated, influencing sensitivity to short versus sustained artifacts (Kothe & Jung, 2016; Chang et al., 2018). Baseline EEG sessions from 39 Rett patients (N = 39) were included in the parameter evaluation. As shorter windows may better capture low-frequency characteristics typical of pediatric EEG (Somervail et al., 2023), the difference in partial correlation coefficients between the automated and original pipelines—for the known relationship between delta (1–4 Hz) power and the Revised Motor Behavioral Assessment (RMBA) score controlling for age—was used as the decision criterion (Raspa et al., 2020; Saby et al., 2024). Optimal parameters were defined as those yielding the highest positive Δr value, indicating maximal enhancement of the expected brain–behavior correlation. The grid of Δr values was visualized as a heatmap to illustrate parameter sensitivity (Supplementary Figure 2).

Evaluating the correlation between delta (1–4 Hz) power and RMBA scores controlling for the effect of age across parameter sweeps revealed a trade-off between aggressive artifact removal and neural signal retention. Increasing the burst criterion from 5 to 25 tended to enhance the correlation effect when shorter ASR window lengths (0.5–1 s) were used, whereas larger windows (≥ 4 s) did not show a consistent dependence on the burst criterion. Changes in ASR window length alone did not produce a monotonic trend for a fixed burst criterion; however, the two parameters interacted such that higher burst criteria combined with shorter windows yielded the largest improvements. The optimal ASR configuration was identified as a burst criterion of 25 and a window length of 0.5 s, which produced a modest but positive increase in the correlation coefficient (Δr = 0.024) relative to the original pipeline (Supplementary Figure 2).

## Polynomial Model Selection for the Model-Based Inflection Approach

To characterize the relationship between the modified coefficient of variation (mCV) and cumulative epoch length, Generalized Estimating Equation (GEE) models were fit using polynomial functions of increasing degree. The objective was to identify the lowest-order polynomial that sufficiently captured the nonlinear decline in mCV while avoiding overfitting.

Following recommendations by Shults and Hilbe (2014), we evaluated polynomial degrees 1 through 4; higher orders were not considered because they can introduce oscillatory artifacts, reduce interpretability, and provide negligible improvement for longitudinal trend modeling. For each frequency band, GEE models were estimated with exchangeable working correlation structure, robust (sandwich) standard errors, mCV as the dependent variable, and epoch number as the polynomial term.

Model adequacy was evaluated using six validated goodness-of-fit criteria: [1] qicp (quasi-likelihood information criterion; Pan, 2001), [2] qicA (dimension-adjusted qicp; Shults & Hilbe, 2014), [3-5] RJ1, RJ2, and DBAR (Rotnitzky–Jewell criteria; Rotnitzky & Jewell, 1990), and [6] cicp (second term of qicp, proposed by Hin & Wang, 2009)

Lower values indicate better fit for all indices. For each polynomial order and frequency band, these metrics were computed and summarized in Supplementary Table 1. The best-fitting polynomial was defined as the degree that minimized the largest number of criteria, prioritizing parsimony when fit indices were similar across adjacent models. In all bands, the fourth-degree polynomial provided the best overall fit and was therefore used to compute the first-derivative inflection point defining the Model-Based Inflection stability threshold.

Goodness-of-fit results for polynomial degrees 1–4 are shown in Supplementary Table 1. Across all five canonical frequency bands (delta, theta, alpha, beta, gamma), the fourth-degree polynomial consistently minimized the majority of fit criteria, indicating superior model adequacy compared to lower-degree alternatives. These results support the use of fourth-degree models for estimating data-length inflection points in the Model-Based Inflection Approach.

# Figures and Tables

## Supplementary Figures


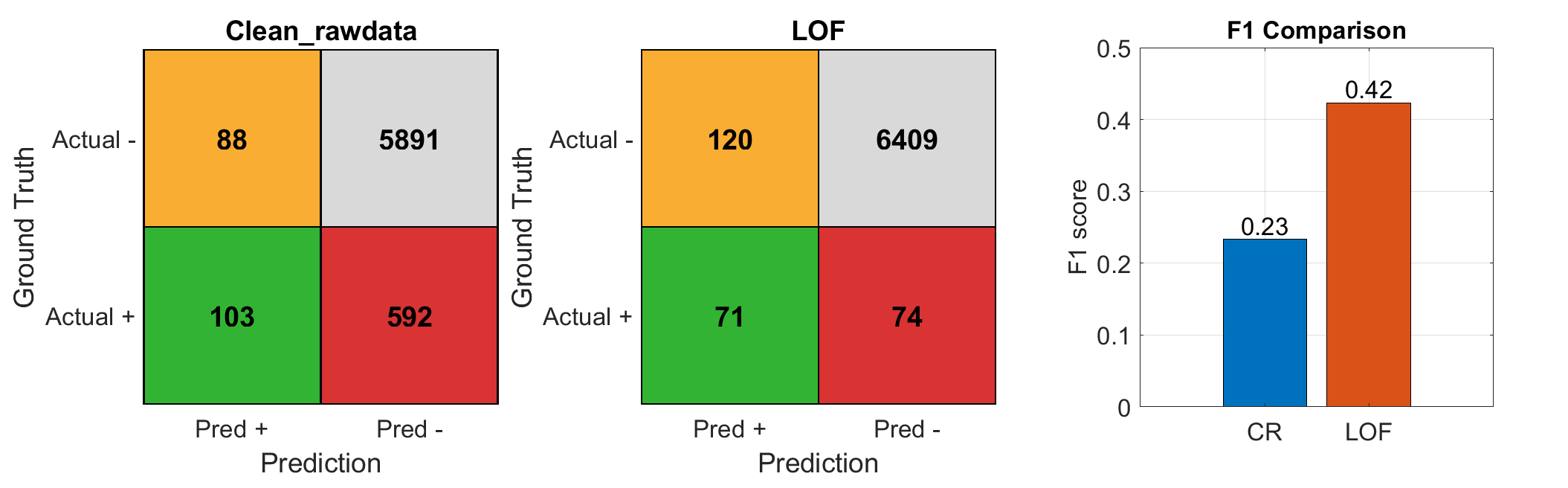


**Supplementary Figure 1.** Confusion matrix and bar plot of F1 score from automated channel rejection performance. Channel rejection algorithm in clean_rawdata (CR) plugin (Green: True-Positive, Red: Orange: False-Positive, Red: False-Negative, Gray: False-Negative)


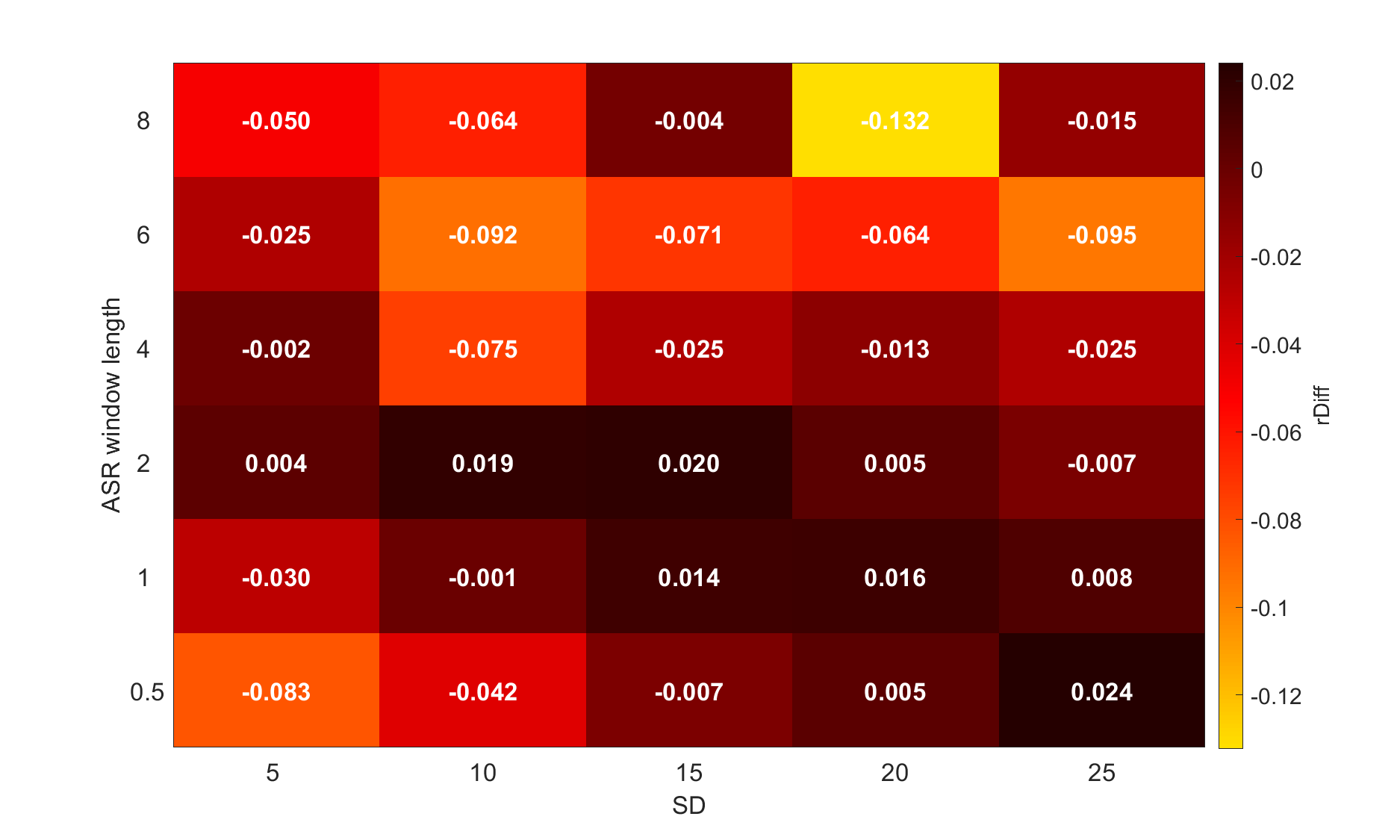


**Supplementary Figure 2.** Heatmap showing the difference in correlation coefficients between delta power and RMBA scores across ASR burst criterion and window-length parameters. Warmer colors indicate greater enhancement of the expected brain–behavior correlation. The optimal ASR parameter set (burst criterion = 25, window length = 0.5 s) yielded the highest positive Δr (0.024).

## Supplementary Table

| Band | Order | qicp | qicA | RJ1 | RJ2 | DBAR | cicp |
| --- | --- | --- | --- | --- | --- | --- | --- |
| Delta | 1 | 226.9 | 227.1 | 11.4 | 282.3 | 259.6 | 58.2 |
|  | 2 | 242.8 | 243.1 | 12.1 | 298.4 | 274.1 | 66.9 |
|  | 3 | 252.6 | 253.0 | 11.2 | 270.6 | 248.1 | 72.5 |
|  | 4 (Best) | 215.4 | 215.9 | 9.5 | 203.8 | 184.9 | 54.5 |
| Theta | 1 | 250.7 | 251.0 | 12.7 | 348.7 | 323.3 | 61.7 |
|  | 2 | 265.6 | 266.0 | 13.1 | 331.6 | 305.3 | 70.3 |
|  | 3 | 274.8 | 275.3 | 12.0 | 308.4 | 284.3 | 75.8 |
|  | 4 (Best) | 229.5 | 229.9 | 9.1 | 184.7 | 166.5 | 53.6 |
| Alpha | 1 | 278.0 | 278.3 | 12.9 | 357.8 | 332.1 | 63.9 |
|  | 2 | 295.1 | 295.4 | 14.0 | 349.7 | 321.6 | 72.9 |
|  | 3 | 304.4 | 304.8 | 12.7 | 316.6 | 291.2 | 77.9 |
|  | 4 (Best) | 256.9 | 257.4 | 9.1 | 183.7 | 165.5 | 54.5 |
| Beta | 1 | 326.5 | 326.7 | 14.2 | 432.6 | 404.3 | 66.6 |
|  | 2 | 341.7 | 342.1 | 14.6 | 401.3 | 372.2 | 74.8 |
|  | 3 | 351.8 | 352.2 | 13.4 | 363.1 | 336.4 | 80.1 |
|  | 4 (Best) | 302.1 | 302.6 | 10.1 | 223.1 | 203.0 | 55.5 |
| Gamma | 1 | 355.4 | 355.6 | 13.5 | 394.7 | 367.7 | 67.4 |
|  | 2 | 373.4 | 373.7 | 15.5 | 410.7 | 379.7 | 77.3 |
|  | 3 | 383.1 | 383.6 | 14.2 | 385.7 | 357.3 | 82.6 |
|  | 4 (Best) | 336.7 | 337.1 | 10.1 | 221.0 | 200.9 | 59.8 |

**Supplementary Table 1.** Goodness-of-fit criteria for polynomial models used to estimate the relationship between mCV and epoch length. This table reports six goodness-of-fit indices (qicp, qicA, RJ1, RJ2, DBAR, and cicp) for GEE models of polynomial order 1–4, evaluated separately for each frequency band. Lower values indicate better model fit. For each fit criterion within each frequency band, the lowest (best) value is highlighted in bold. The “Best” row marks the polynomial order that minimized the greatest number of criteria and was therefore selected as the optimal model. Polynomial degrees higher than four were not considered to avoid overfitting. Brief descriptions of each criterion (qicp, qicA, RJ1, RJ2, DBAR, cicp) are provided in the Supplementary Methods.
